# Uterine glands impact uterine receptivity, luminal fluid homeostasis and blastocyst implantation

**DOI:** 10.1038/srep38078

**Published:** 2016-12-01

**Authors:** Andrew M. Kelleher, Gregory W. Burns, Susanta Behura, Guoyao Wu, Thomas E. Spencer

**Affiliations:** 1 Division of Animal Sciences, 158 ASRC, 920 East Campus Drive, University of Missouri, Columbia 65211, USA; 2 Department of Animal Science, Faculty of Nutrition, Texas A&M University, College Station 77843-2471, USA

## Abstract

Uterine glands are essential for pregnancy in mice and likely humans, because they secrete or transport bioactive substances that regulate uterine receptivity for blastocyst implantation. In mice, the uterus becomes receptive to blastocyst implantation on day 4, but is refractory by day 5. Here, blastocysts could be recovered from progesterone-induced uterine gland (PUGKO) but not wildtype (WT) mice on day 5 post-mating. Anti-adhesive Muc1 protein and microvilli were present on the luminal epithelium of PUGKO but not WT uteri. A number of known uterine receptivity genes and gland-specific genes were altered in the PUGKO uterus. Next, the uterus and uterine luminal fluid (ULF) were obtained from WT and PUGKO mice on day 3, 4 and 5. Transcriptome analysis revealed that 580 genes were decreased in the PUGKO uterus, however ULF secrotome analysis revealed that many proteins and several amino acids were increased in the PUGKO ULF. Of note, many proteins encoded by many gland-specific genes were not identified in the ULF of WT mice. These results support the ideas that uterine glands secrete factors that regulate ULF homeostasis and interact with other cell types in the uterus to influence uterine receptivity and blastocyst implantation for the establishment of pregnancy.

In humans and rodents, implantation involves blastocyst apposition, attachment, and adhesion to the luminal epithelium (LE) followed by infiltration and growth of the trophectoderm into the decidualizing stroma^1^. This complex process requires an intricate dialogue between an implantation-competent blastocyst and a receptive uterus^2^. Uterine receptivity can be defined as the state of the endometrium when it is ready to support blastocyst implantation and establishment of pregnancy. In humans, the inability of the uterus to achieve a receptive status is likely a major cause of infertility^3^,^4^,^5^. In mice, blastocysts enter the uterus by the morning of gestational day (GD) 4, and the endometrium becomes receptive by the afternoon of day 4^2^,^6^. By the afternoon of GD 5, the uterus is non-receptive and refractory to blastocyst implantation^7^. The implantation process is initiated by blastocyst trophectoderm apposition and attachment to the LE on GD 4. By GD 5, the LE cells at the implantation sites undergo apoptosis or entosis, allowing the motile blastocyst trophectoderm to contact stromal cells and initiate decidualizations^8^,^9^. Stromal cell decidualization is important for regulating blastocyst implantation and formation of a functional placenta^10^.

Uterine function is primarily regulated by the ovarian steroid hormones estrogen and progesterone^11^. Under their influence, the LE, glandular epithelia (GE), and stroma express genes important for uterine receptivity and blastocyst implantation, including cytokines, growth factors, and lipid mediators^2^,^12^,^13^,^14^. The phenotype of mouse models lacking uterine glands emphasized the importance of endometrial glands and, by inference, their secretions for blastocyst implantation^15^,^16^,^17^,^18^,^19^. Deficient glandular activity, usually described as a secretory phase defect, is hypothesized to be a significant underlying cause of early pregnancy failure in women^3^,^7^. Secretions present in the uterine lumen of mice are not well characterized, but likely contain amino acids, glucose, proteins, and other substances based on studies of uterine luminal fluid (ULF) in humans and domestic animals^20^,^21^,^22^,^23^,^24^. The first GE-derived factor found to be essential for pregnancy in mice was leukemia inhibitory factor (LIF). *Lif* is expressed in the GE in response to the nidatory surge of estrogen from the ovary on GD 3.5, and *Lif* null mice are infertile and exhibit defects in uterine receptivity, blastocyst implantation and stromal cell decidualization^25^. Additional studies of several mouse models lacking uterine glands provide further evidence that the GE and their secretions have important biological roles in blastocyst implantation and stromal cell decidualization^17^,^18^,^26^,^27^,^28^. However, how uterine glands regulate those fundamental processes important for the establishment and maintenance of pregnancy is not well understood.

A series of studies are presented here to further understand the infertility of progesterone-induced uterine gland knockout (PUGKO) mice and contributions of the endometrial GE to the uterine transcriptome and ULF secretome. PUGKO mice are generated by exposing neonatal C57BL/6 mice to progesterone from postnatal days 2 to 10^18^,^29^. That exposure regimen permanently inhibits differentiation of GE in the uterus, which normally beings during the second week of life, but does not affect the differentiation of other reproductive tract structures such as the ovary, oviduct, vagina or cervix. The resulting mice have normal estrous cycles as adults, but are infertile and exhibit defects in blastocyst implantation and stromal cell decidualization^17^,^18^,^19^. Results of these studies support the ideas that uterine glands impact uterine gene expression and ULF homeostasis and play an important role in uterine receptivity and blastocyst implantation for establishment of pregnancy.

## Results

### PUGKO mice lack endometrial glands

**Figure 1:**
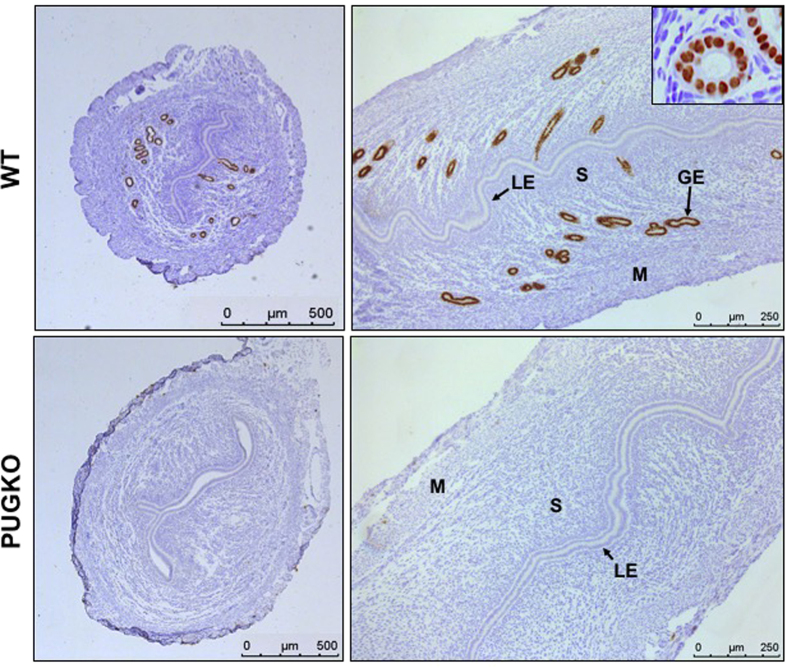
Expression of FOXA2 in the uterus. Immunohistochemical localization of FOXA2 protein in uteri of control (WT) and PUGKO mice on day of pseudopregnancy (DOPP) 4. Cross (right panels) and longitudinal (left panels) uterine sections were counterstained with hematoxylin. Note nuclear localization of FOXA2 (inset panel B). GE, glandular epithelium; LE, luminal epithelium; S, Stroma. Scale bars = 500 μm for left panels and 250 μm for right panels.

Forkhead box A2 (FOXA2) is a transcription factor that is expressed solely in the GE of both neonatal and adult mouse uteri^27^,^30^. In day of pseudopregnancy 4 (DOPP 4) wildtype (WT) mice, immunoreactive FOXA2 protein was detected in the nuclei of GE cells in the endometrium and was not present in other uterine cell types (LE, stroma or myometrium) (Fig. 1). In contrast, uteri from DOPP 4 PUGKO mice were completely devoid of immunoreactive FOXA2 protein. With the exception of glands, there was no consistent morphological or histological differences between the adult WT and PUGKO uteri.

### Embryo implantation is defective in PUGKO mice

**Figure 2:**
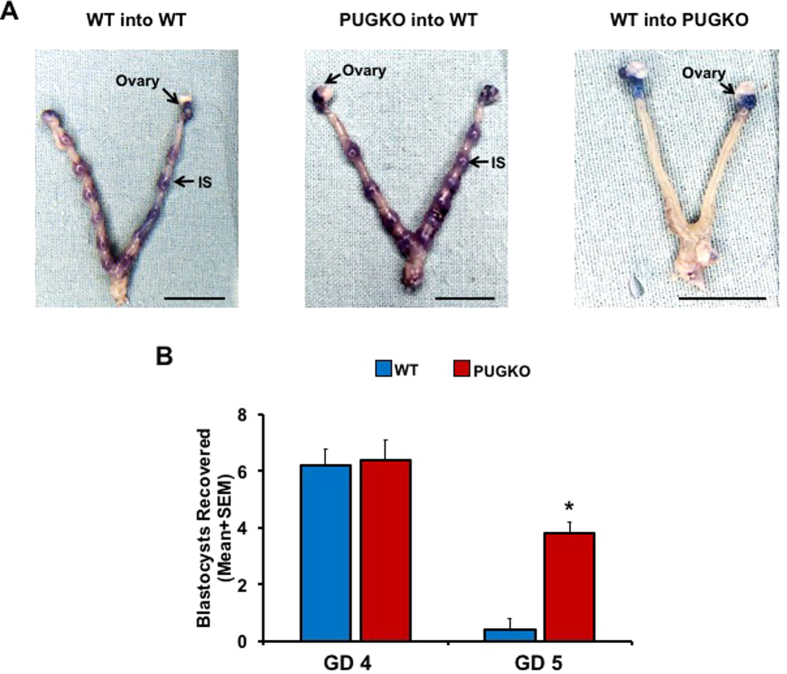
Blastocyst attachment and implantation. (**A**) Outcome of embryo transfer between wildtype (WT) and PUGKO mice. Embryo implantation sites (IS) were visualized by tail vein dye injection on gestational day 6 after transfer on day 3. No difference in number or size of embryo implantation sites were observed between WT mice receiving WT or PUGKO embryos. Scale bar = 1 cm. (**B**) Blastocysts recovered in uterine flush of wildtype (WT) and PUGKO mice on gestational days 4 and 5 at 1600 h. The asterisk (*) denotes differences (P < 0.05) between WT and PUGKO mice.

A reciprocal embryo transfer experiment was performed between WT and PUGKO mice. Both WT and PUGKO mice were mated to intact males, and blastocysts were recovered from the uterine lumen on GD 4. Blastocysts from WT mice were transferred into DOPP 3 WT or PUGKO recipient mice, and blastocysts were also transferred from PUGKO mice into DOPP 3 WT recipient mice. Blastocyst implantation sites were visualized 72 hours later (GD 6) by intravenous injection of Evans blue dye, which accumulates at sites of embryo implantation due to increased vascular permeability^31^. Implantation sites were observed in WT recipient mice receiving blastocysts from either WT or PUGKO mice (Fig. 2A). The number of implantation sites observed did not differ between WT recipients receiving WT blastocysts and those receiving PUGKO blastocysts (P > 0.05). An average of 8 (range 5–11) and 7.6 (range 4–10) implantations were observed in WT recipients of WT blastocysts and those receiving PUGKO blastocysts, respectively. Further, the diameter of the implantation sites was not different (P > 0.05) in WT recipients of WT blastocysts (2.8 ± 0.2 mm) and those receiving PUGKO blastocysts (2.5 ± 0.1 mm). Blastocyst implantation sites were not observed in any PUGKO recipient mouse receiving WT blastocysts (Fig. 2A).

By the afternoon of GD 5, blastocysts are firmly attached to the LE within implantation crypts and can no longer be flushed from the uterus^32^. Both WT and PUGKO mice were mated to males of proven fertility, and the uterus was gently flushed on the afternoon of GD 4 or 5 (GD 1 = observation of vaginal plug) (Fig. 2B). On GD 4, blastocysts were recovered from both WT and PUGKO mice. In contrast, blastocysts could be recovered in the uterine flush from PUGKO but not WT mice on GD 5. These results support the idea that the blastocyst fails to attach or adhere to the uterine LE in PUGKO mice.

### Uterine receptivity is defective in PUGKO mice

**Figure 3:**
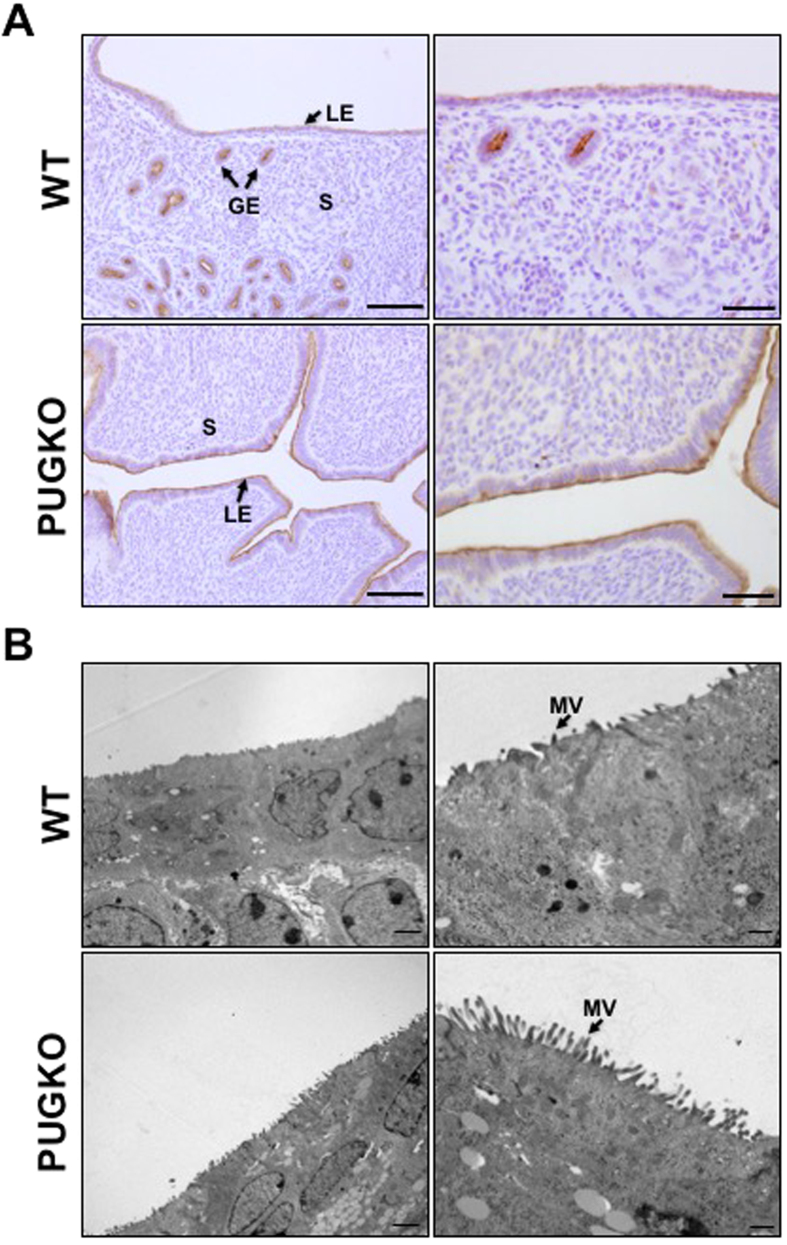
MUC1 and microvilli in the uterus. (**A**) Immunohistochemical localization of MUC1 in wildtype (WT) and PUGKO uteri on DOPP 4. Note the absence of MUC1 on the luminal epithelia of WT but not PUGKO uteri. Sections were counterstained with hematoxylin. Abbreviations: GE, glandular epithelium; LE, luminal epithelium; S, stroma. Scale bars = 100 μm for left panels and 50 μm for right panels. (**B**) Transmission electron microscopy analysis of the uterine luminal epithelium from wildtype (WT) control and PUGKO uteri on DOPP 5 at 800× (left panels) and 2500× magnification (right panels). Note the increase number and length of microvilli in the PUGKO mice. Abbreviations: MV, microvilli; S, stroma. Scale bars = 2 μm for left panels and 0.5 μm for right panels.

Expression of the anti-adhesive glycoprotein mucin one (MUC1) on the LE is considered an important indicator of uterine receptivity^33^. As the uterus achieves receptivity, MUC1 expression is decreased on the LE between GD 3 and 4, and persistent MUC1 is indicative of a nonreceptive uterus. In uteri of WT mice on the afternoon of DOPP 4, immunoreactive MUC1 protein was abundant on the apical surface of the GE, but absent from the LE (Fig. 3A). In contrast, MUC1 protein remained abundant on the LE of uteri from PUGKO mice.

Another important parameter for acquisition of uterine receptivity is membrane transformation of the uterine LE. The presence of long microvilli on the LE apical surface indicates a lack of receptivity, as a marked flattening of these microvilli occurs prior to implantation^34^. Transmission electron microscopy (TEM) revealed that, in contrast to WT uteri, the LE of PUGKO uteri fail to undergo microvilli flattening, indicating impaired uterine receptivity (Fig. 3B). As illustrated in Fig. 3B, the number and length of microvilli (MV) were substantially greater on the apical surface of the LE of PUGKO uteri. Microvilli were almost two-fold longer (P < 0.05) on the LE of PUGKO (0.77 ± 0.04 μm) as compared to WT (0.40 ± 0.04 μm) uteri. The persistent MUC1 protein and lack of membrane transformation clearly indicate that the LE of the PUGKO uterus is not receptive to blastocyst implantation.

**Figure 4:**
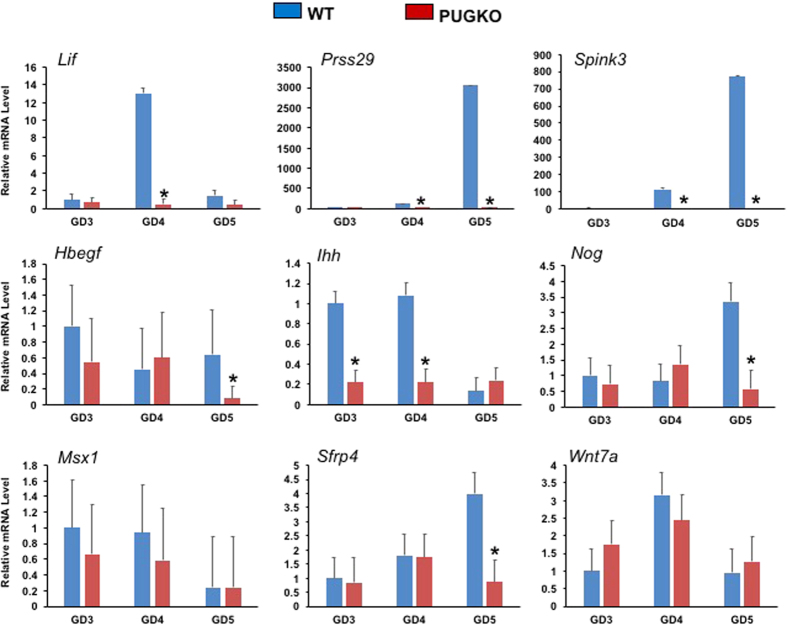
Relative expression of uterine receptivity genes in pregnant mice. Relative expression levels (2^−ΔΔCT^) were determined by quantitative RT-PCR. Data were analyzed by the comparative C_T_ method using *Gapdh, Hprt* and *Actb* as the internal control. Data are presented as fold change relative to DOPP 3 WT uteri. The asterisk (*) denotes differences (P < 0.05).

Uterine receptivity in mice also requires dynamic changes in gene expression in the uterine epithelia as well as stroma^2^,^6^,^35^. Receptivity and implantation-associated genes that are expressed in the LE (*Hbegf, Ihh, Msx1, Sfrp4, Wnt7a*), GE (*Lif, Ihh, Msx1, Prss29, Spink3*), LE and GE (*Ihh, Msx1)*, and/or stroma (*Nog, Sfrp4*) were quantified in the uteri from bred WT and PUGKO mice by qPCR (Fig. 4). The abundance of GE-expressed (*Ihh*) and GE-specific genes (*Lif, Prss29, Spink3*) were substantially reduced or absent (P < 0.05) in the PUGKO uterus. Expression of *Hbegf, Nog* and *Sfrp4* were also decreased (P < 0.05) in the PUGKO uterus on GD 5. In contrast, expression of *Msx1* and *Wnt7a* were not different (P > 0.05) (Fig. 4).

### Transcriptomic analysis of uterine receptivity in pseudopregnant mice

**Figure 5:**
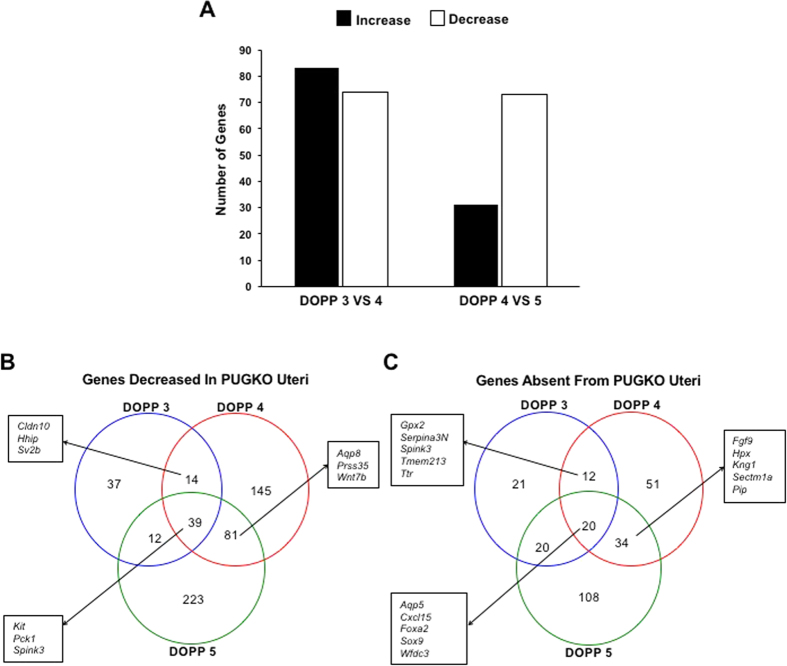
RNA sequencing analysis of uteri from pseudopregnant mice. (**A**) Number of genes that increase and decrease in the uterus of WT mice from DOPP 3 to 4 and DOPP 4 to 5. (**B**) Venn diagram illustrating genes that decrease (P < 0.05) in PUGKO uteri on DOPP 3, 4, and 5. (**C**) Venn diagram illustrating genes that are absent (P < 0.05, RPKM < 1) in PUGKO uteri on DOPP 3, 4, and 5.

Pseudopregnant mice experience the same changes in uterine gene expression as early pregnant mice^6^, and samples of the ULF can be obtained without blastocysts^36^. Therefore, uteri were obtained from WT and PUGKO mice on DOPP 3 (pre-receptive), 4 (receptive), and 5 (post-receptive). The lumen of each uterus was gently flushed with PBS to obtain ULF, and the uterus and ULF were frozen for subsequent analysis. First, total RNA was extracted from the uteri and sequenced. Transcriptome analysis revealed dynamic changes in gene expression in WT mice (Fig. 5A and Supplemental Table 1). Expression of 157 genes (83 increased, 74 decreased) changed (P < 0.05) from DOPP 3 to 4 (Fig. 5B and Supplemental Table 1). The top 10 protein coding genes increased from DOPP 3 to 4 were *Prss28, Prss29, 8430408G22Rik, Pip, Atp6v0d2, Lrat, Prss35, Cyp26a1, Guca2b,* and *Slc46a2* (Table 1). Of note, *Prss28* and *Prss29* are only expressed in the GE of the uterus^30^,^37^. The top 10 protein coding genes decreased from DOPP 3 to 4 were *BC048679, Clca3, Sprr2f, Ltf, Ppp1r1b, Gstm7, Rdh1, Sprr2g, Gpr128, Gcnt,* and *S100a9* (Table 1). Between DOPP 4 and 5, expression of 104 genes (31 increased, 73 decreased) changed (P < 0.05) in the WT uterus (Fig. 5B and Supplemental Table 1). The top 10 protein coding genes increased from DOPP 4 to 5 were *Gm10263, Gm10260, Gtsf1, Glp1r, H2-q10, Gm21830, Hyal3, Gpr81, C130074G19Rik,* and *Armcx4* (Table 2). The top 10 protein coding genes decreased from DOPP 4 to 5 were *Rps2-ps13, Gm26619, Gm102063 Gm10260, H2-Ea-Ps, Gm12328, H2-Bl, RP23-184F1.2, Gtsf1,* and *Glp1r* (Table 2).

**Figure 6:**
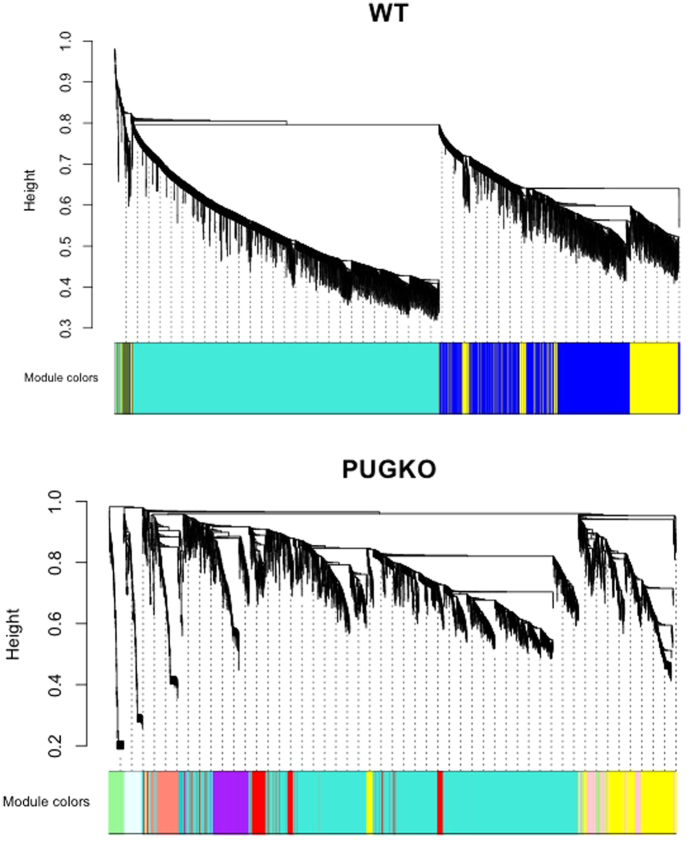
Weighted gene correlation networks of genes. The cluster dendrograms show modular expression patterns of genes in varying number of modules between control and PUGKO mouse. The upper panel shows the cluster tree of gene expression based on unassigned topology overlapping generated from the expression data. The lower panels show the corresponding expression modules (in different colors). The vertical axis represents the branch height of cluster trees. In both WT and PUGKO, a threshold of branch height for 0.2 was applied to merge modules (modules whose Eigengenes are correlated above 0.8 were merged). The adjacency matrix for topology overlapping was calculated based on a soft-thresholding power of 6. The minimum module size used was 30. No reassignment of thresholds was allowed in detecting the modules.

Hierarchical clustering based on weighted gene co-expression network analysis identified an extensive modular network pattern of genes in WT uteri that substantially differed in PUGKO uteri (Fig. 6). The present study identified 21 of the top 25 differentially expressed genes (i.e., *Spink3, Cxcl15, Cyp3a25, Tmprss11g*) previously identified in the uteri of WT and PUGKO mice on DOPP 4 by microarray^37^. Here, the expression of 1,210 genes differed in uteri of WT and PUGKO mice from DOPP 3 to 5 (Supplemental Table 2). Expression of 444 genes were more abundant and 580 genes less abundant in PUGKO as compared to WT uteri (Fig. 5B). Of the 580 decreased genes, 253 genes were not expressed (RPKM < 1) in the PUGKO uterus (Fig. 5C). Many of those 253 genes are exclusively expressed in the GE (i.e., *Cxcl15, Foxa2, Sox9, Spink3*) or abundantly expressed in the GE but also expressed in other cell types (i.e., *Fgf9, Lrat, Prss35*)^2^,^30^. Integration with uterine cell specific transcriptomic data previously published by our lab^37^ illustrated that 24% and 29% of the genes absent from the PUGKO uterus on DOPP 3 and 4, respectively, were enriched in the GE of WT mice. Expression of 26 of the 253 absent genes in the PUGKO uterus (i.e., *Prss28, Prss29, Pip, Serpina3n*) increased (P < 0.05) between DOPP 3 to 4 in WT mice, whereas expression of only 4 of the 253 genes *(H2-Ea-ps, H2-Q10, Hyal3, Gpr81)* increased (P < 0.05) from DOPP 4 to 5 in WT mice (Supplemental Table 1).

Bioinformatics analyses, including gene ontology (GO) and pathway analyses, were conducted on differentially expressed genes to predict functionally impacted pathways. Genes with decreased expression in the PUGKO compared to WT uterus were largely associated with cellular and stimulus-response related activities (Table 3). The genes expressed in WT but not in PUGKO uteri are mostly enriched in gene ontologies associated with metabolism (Table 3). Expression changes of the two gene sets were further analyzed for scoring differential regulation of pathways using a method that incorporates pathway topology and the magnitude of gene expression changes^38^. Genes expressed in WT but not PUGKO uteri, were associated with pathways such as complement and coagulation cascades (Supplementary Figure 1), different amino acid metabolic pathways, Wnt signaling, and dopaminergic synapses. Genes with decreased expression in PUGKO compared to WT uteri are associated with pathways including cell cycle and amino acid and nucleotide metabolism. Specific pathways such as synapse pathways (serotonergic and dopaminergic), cell adhesion and tryptophan metabolism are commonly associated with both differentially expressed gene sets. Furthermore, subsets of the differentially expressed genes are associated with bicluster expression patterns (similar expression changes by subset of genes among subset of samples), suggesting tightly coordinated local cross-talk among the differentially expressed genes. The global cross-talk among all the discovered differentially expressed genes displayed topological variations between WT and PUGKO uteri (Supplementary Figure 2), which reaffirms alterations in transcriptional networks of genes in the uteri of PUGKO compared to WT mice (Fig. 6).

### Proteins in the ULF

Uterine GE synthesize and secrete or transport bioactive substances, including proteins, amino acids and glucose, into the uterine lumen^17^,^18^,^19^,^27^,^28^. Proteins in the ULF of DOPP 3, 4 and 5 WT and PUGKO mice were identified and quantified using mass spectrometry. A total of 1,359 proteins were detected in WT ULF based on spectral count data (Supplemental Table 3). As summarized in Table 4, the most abundant proteins detected in WT ULF on all DOPP were serum proteins including albumin (Alb), alpha 2 macroglobin (A2m), and complement C3 (C3). Of particular note, several secreted proteins known to be expressed only by the GE of the uterus (e.g., LIF, PRSS28, PRSS29, SPINK3) were not found in the ULF of WT mice.

**Figure 7:**
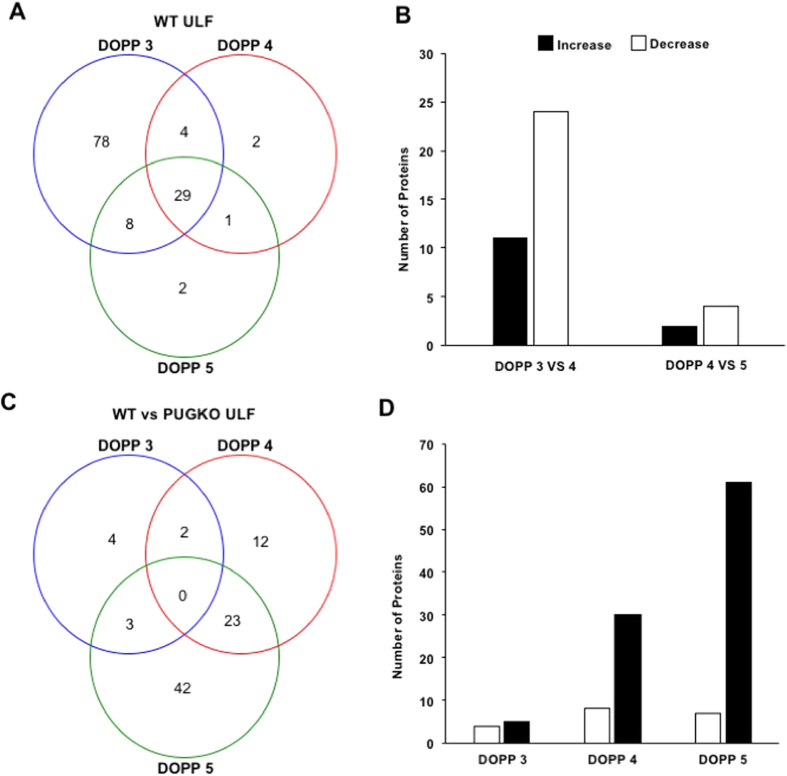
Proteins in the uterine lumen. (**A**) Proteins identified in the WT ULF on DOPP 3, 4 and 5. (**B**) Bar chart of the number of proteins that increase and decrease from DOPP 3 to 4 and DOPP 4 to 5 in the WT ULF. (**C**) Number of proteins with different abundances in the PUGKO ULF compared to WT. (**D**) Bar chart of the number of proteins that increase and decrease in the PUGKO ULF compared to WT DOPP 3 to 4 and DOPP 4 to 5.

Further analyses of the proteomics data considered proteins with a minimum of 4 unique spectral counts in 4 of the 5 ULF samples. Using that criteria, WT mice ULF contained 120 proteins on DOPP 3, 36 proteins on DOPP 4, and 40 proteins on DOPP 5 (Fig. 7A and Supplemental Table 4). Integration of uterine transcriptome and ULF proteome data from WT mice found that 100 of the 124 ULF proteins are encoded by genes expressed in the uterus. Thus, 24 of the ULF proteins originate from serum such as albumin and hemoglobin. Between DOPP 3 and 4 in WT mice, the abundance of 26 proteins changed (P < 0.05) in the ULF (12 increased, 24 decreased) (Fig. 7B). In contrast, only 6 proteins changed from DOPP 4 to 5 (2 proteins increased, 4 decreased) in WT ULF (Fig. 7B and Supplemental Table 4).

Unexpectedly, the ULF of PUGKO mice contained more proteins, particularly on DOPP 4 and 5, compared to WT ULF (Fig. 7C and D and Supplemental Table 5). In fact, few proteins were less abundant in the ULF of PUGKO as compared to WT mouse uteri, including 4 proteins on DOPP 3 (CALB1, HBA1, KRT19, KRT9), 8 proteins on DOPP 4 (APOE, HMGB1, SERPINA3K, SOD1, TMSB10, TMSB4X, TPM4, VCL), and 7 proteins on DOPP 5 (HMGB1, SOD1, VIM, FASN, HSPB1, TMSB10, TPM4) (Supplemental Table 4). Of those, only 12 proteins (APOE, CALB1, FASN, HMGB1, KRT19, SOD1, TMSB10, TMSB4X, TPM4, VIN) were encoded by genes expressed in the uterine GE based on transcriptomic data.

### Select nutrients in the ULF

**Figure 8:**
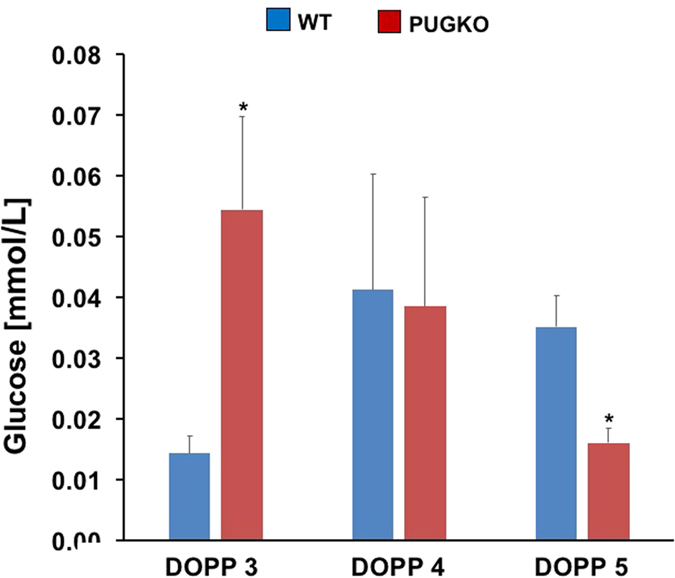
Glucose in the uterine lumen. Glucose was quantified in samples of ULF from WT and PUGKO mice on DOPP 3, 4 and 5 (n = 5 mice per day and type) using a fluorometric glucose assay kit. Comparison of the glucose present in the ULF of WT and PUGKO mice on DOPP 3, 4 and 5. Asterisk (*) denotes differences (P < 0.05).

Glucose is an important energy source required for development to the blastocyst stage and may regulate trophectoderm activation for implantation in mice^39^,^40^. As shown in Fig. 8, glucose concentrations increased (P < 0.05) in the ULF of WT mice from DOPP 3 to 4 and remained elevated on DOPP 5. In contrast, glucose concentrations were higher on DOPP 3 and lower on DOPP 5 (P < 0.05, day x type) in the ULF of PUGKO mice. Transcriptomic analysis of the glycolytic and gluconeogenesis pathway found differences in only two solute carriers (*Slc2a1* and *Slc2a3*) on DOPP 5. Interestingly, phosphoenolpyruvate carboxykinase 1 (*Pck1*), a major control point in gluconeogenesis, is decreased in the PUGKO uterus during the peri-implantation period. Further, *Pck1* was previously identified as a GE enriched gene on DOPP 4^37^.

**Figure 9:**
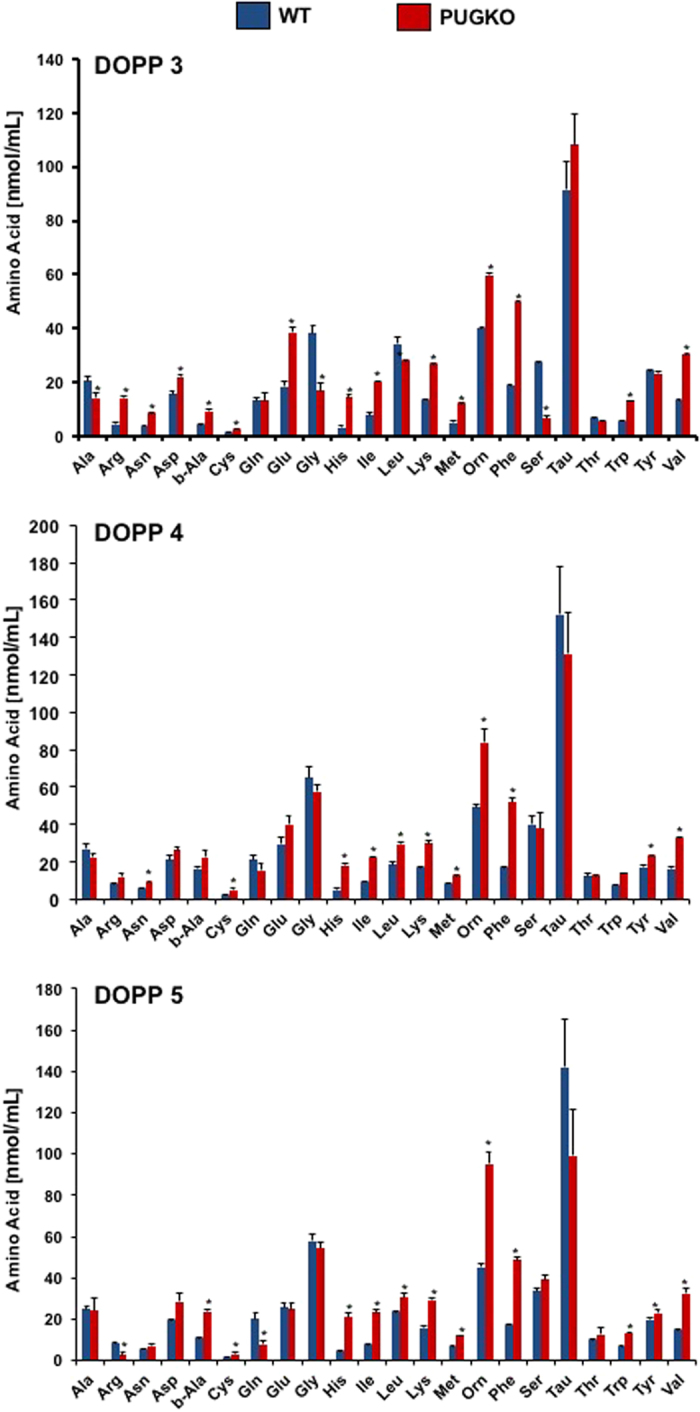
Amino acids in the uterine lumen. Amino acids, except for proline, were determined in samples of ULF from WT and PUGKO mice on DOPP 3, 4 and 5 (n = 5 mice per day and type) by fluorometric HPLC (**A**) Comparison of the amino acid profile in the ULF of WT and PUGKO mice on DOPP 3, (**B**) DOPP 4, and (**C**) DOPP 5. Asterisk (*) denotes differences (P < 0.05).

Select amino acids are also important for blastocyst implantation, and those nutrients are transported into the uterine lumen by solute carrier transporter proteins^41^,^42^. Trophectoderm outgrowth of the mouse blastocyst *in vitro* and likely implantation *in vivo* requires amino acids. Concentrations of amino acids in the ULF of WT mice changed (P < 0.05, day) from DOPP 3 to 5 (Table 5), and Glu, Asn, Ser, Gln, Gly, Thr, Cit, Arg, b-Ala, Tau, Trp, Met, Val, Orn, and Lys increased from DOPP 3 to 4. Many amino acids were more abundant (P < 0.05) in PUGKO than WT ULF on DOPP 3, 4 and 5 (Fig. 9). None of the amino acids were lower (P > 0.10) in the ULF of PUGKO as compared to WT ULF on DOPP 4, but Ser was lower (P < 0.05) on DOPP 3, and Gln and Arg were lower on DOPP 5 (Fig. 9).

## Discussion

The present study confirmed that progesterone treatment permanently ablates the differentiation of GE in the neonatal uterus, resulting in an adult uterus that lacks detectable FOXA2 protein and thus glands. Mouse models with substantially reduced or absent uterine glands exhibit a subfertile or infertile phenotype due to defects in blastocyst implantation^17^,^18^,^26^,^27^,^28^,^43^,^44^. In the present study, morphologically normal blastocysts were recovered from the PUGKO uterus on GD 4 and 5, and GD 4 blastocysts from PUGKO mice could implant when transferred into the uterus of WT mice. Thus, the pituitary, ovary, oviduct, and vagina appear to function normally in PUGKO mice. Our previous study found that spatiotemporal patterns of steroid receptor expression (ESR1, PGR) and several steroid hormone-regulated genes were not altered in the uterus of pseudopregnant PUGKO mice^17^. Thus, the defects in blastocyst implantation in PUGKO mice can be ascribed to inadequate uterine receptivity and/or blastocyst trophectoderm attachment and motility, which are critical processes in implantation for establishment of pregnancy in mice and humans. Results from the present studies clearly support the idea that the blastocyst trophectoderm is unable to attach and adhere to the uterine LE of PUGKO mice, since blastocysts could be recovered by flushing the uterine lumen of PUGKO but not WT mice on GD 5. In mice, structural and molecular changes in the uterine LE occur between GD 3 and 4 that render it receptive to blastocyst implantation^2^. The first steps in implantation involve the apposition and adhesion of the blastocyst trophectoderm to the uterine LE. The removal of anti-adhesive MUC1 protein from the glycocalyx of the LE prior to implantation facilitates interaction of the trophectoderm with the LE^45^. Microvilli also become less numerous and pronounced as the LE cells lose polarity^46^. Thus, the uterine LE failed to undergo changes associated with development of receptivity in PUGKO mice, because persistent MUC1 protein and microvilli were observed on the LE. A similar phenotype was reported for the LE in uteri of *Lif* null mice^47^. *Lif* is expressed specifically in the uterine GE in response to the nidatory surge in estrogen from the ovary^48^. The implantation and decidualization defects in *Lif* null mice could be rescued by intraperitoneal injection of a single dose of recombinant LIF 25. Thus, it would be interesting to determine if intraperitoneal injections of recombinant Lif can rescue infertility of glandless mice, such as PUGKO and aglandular *Foxa2* conditional knockout mice. A number of other genes with known or proposed functions in uterine receptivity and decidualization were also reduced or ablated in the PUGKO uterus, including *Ihh, Nog, Prss28, Prss29, Sfrp4* and *Spink3*. Both PUGKO and *Foxa2* conditional knockout mice have defects in stromal cell decidualization based on results of artificial induced deciduoma formation^17^,^27^. Injection of LIF into the uterine lumen was unable to rescue decidualization in PUGKO mice^17^ and only partially rescued decidualization in *Foxa2* conditional knockout mice^27^. In both of those studies, the route of LIF administration may be an important issue given the inability of LIF to be detected in the ULF proteome in the current study.

Transcriptome analysis identified profound changes in uterine gene expression due to the absence of glands, as 444 genes were increased and 580 genes decreased in the PUGKO as compared to WT uterus from DOPP 3 to 5. Bioinformatics analyses revealed that many of the genes decreased or absent in the PUGKO uterus and expressed in the WT uterus are involved in biological processes known or predicted to be important for the establishment of pregnancy, including metabolic processes, transport and response to stimulus. The cell-specific expression and function of the majority of those differentially expressed in the mouse uterus are not known. Of the 580 genes with decreased expression in pseudopregnant PUGKO uteri, a few are known to be expressed solely in the GE including *Cxcl15, Foxa2, Prss28, Spink3,* and *Sox9*^27^,^30^,^37^,^49^. Many of the genes reduced or absent in the PUGKO uterus encode secreted proteins, including LIF, CXCL15, SERPINA3, TMPRSS13, SPRINK3, SERPINB11, PIP, HPX, FGA, MMP7, CALCA, SELENBP1, WNT7B, LYZ, PRSS28, PRSS29. Of particular note, several cytochrome P450 (CYPs) genes (*Cyp26a1, Cyp2f2, Cyp3a16, Cyp3a25, Cyp3a57, Cyp3a59*), proteases (*Mmp7, Prss28, Prss29, Tmprss11a, Tmprss11g, Tmprss13, Tmprss2, Tmprss4*) and solute carriers (Slc) (*Slc12a3, Slc12a6, Slc13a2, Slc1a5, Slc22a4, Slc24a4, Slc26a7, Slc2a1, Slc36a2, Slc39a2, Slc39a4, Slc39a8, Slc44a3, Slc46a2, Slc5a8*) were less abundant in the PUGKO uterus. Cyp genes encode heme‐containing membrane enzymes in the endoplasmic reticulum or mitochondrial inner membrane that regulate hormone and cholesterol synthesis as well vitamin D metabolism and retinoic acid signaling^50^,^51^. Progesterone induces *Cyp26a1* expression in the mouse uterus^52^, and *Cyp26a1* mRNA is higher in the secretory phase than in the proliferative phase endometrium of women^53^.Cyp26a1 is a metabolizing enzyme catalyzing trans-retinoic acid to more polar metabolites^54^. Retinoic acid regulates the differentiation of the uterine epithelium^55^ and the expression of matrix metalloproteases (Mmps)^56^, which are involved in the remodeling of the extracellular matrix of the uterus in preparation for pregnancy^57^. Solute carrier proteins are membrane transporters that control the uptake and delivery of crucial compounds such as glucose and amino acids across epithelia^58^. Several amino acid transporters (*Slc1a1, Slc1a5, Slc36a2*), facilitated glucose transporters (*Slc2a1, Slc2a3*), and sodium/glucose cotransporters (*Slc5a5, Slc5a8, Slc5a11*) were altered in the PUGKO uterus. Glucose metabolism is important for the preparation of the epithelium and stroma for embryo implantation during early pregnancy^40^. Differences in the amount of glucose in the uterine lumen found between WT and PUGKO mice were likely not supraphysiological^24^ and did not affect the ability of day 3 embryos from the PUGKO to implant after transfer to a WT uterus. Amino acid transport is important for blastocyst implantation, and *in vitro* studies found leucine and arginine regulate trophoblast motility in the preimplantation mouse embryo^41^,^42^,^59^. Collectively, the decrease in specific secreted factors, enzymes and transporters in the uterus of PUGKO mice may also contribute to deficient uterine receptivity and defects in blastocyst implantation.

For several decades, the glands of the uterus have been hypothesized to predominantly transport or secrete factors in an apical manner into the uterine lumen and, in that way, regulate survival and growth of the blastocyst for establishment of pregnancy. The present study challenges that idea, because the dynamic changes in uterine gene expression from DOPP 3 to 5 were not reflected in the proteins and amino acids present in the ULF. Indeed, genes altered in the PUGKO uterus are expressed in many different cell types of the uterus in addition to the GE, suggesting that the glands communicate with many different cell types in the uterus. Remarkably, archetypal GE-derived factors (LIF, PRSS28, PRSS29, SPINK3) were not detected in the ULF using mass spectrometry. Although this could be due to technical limitations in terms of sensitivity, we were also unable to detect LIF protein in the ULF of WT mice using a commercially available ELISA assay (data not shown). This result suggests that LIF and other GE-specific genes encoding secreted proteins may be preferentially secreted basally from the glands and act on the stroma and LE or other cells that express Lif receptors (LIFR). Similar to *Lif, Spink3* is also expressed exclusively in GE of the mouse uterus, yet SPINK3 protein was observed in the LE and decidualizing stromal cells as well as uterine glands^49^. Of note, SPINK3 protein was also not detected in the ULF of WT mice in the present study. Collectively, these observations support the idea that the GE synthesizes and secretes many factors basolaterally that act on the stroma and LE to create a unique microenvironment for blastocyst implantation in the antimesometrial area of the uterus where the GE are developmentally located and implantation crypt forms in a successful pregnancy. Future studies are needed to determine the role of the uterine glands and their secretions in paracrine interactions with the LE, stroma and decidual cells^60^.

This study is the first comprehensive investigation of the uterine luminal secretome (glucose, amino acids, proteins) in pseudopregnant or pregnant mice. The results found that dynamic changes in the ULF secretome do not occur in association with the development of uterine receptivity. This finding starkly contrasts with analysis of the ULF secretome during early pregnancy in large animals such as cattle, sheep and pigs^22^,^61^,^62^. Results of the present study to support the idea that uterine GE have a primarily biological role in homeostasis of the ULF, because many proteins and most amino acids were more abundant in ULF from PUGKO than WT mice. Thus, the amino acids (leucine and arginine) and glucose implicated in activation of mouse blastocyst trophectoderm motility and invasiveness were clearly not deficient in the PUGKO uterus. Although the PUGKO mice had detectable differences in ULF protein, amino acids and glucose in DOPP 3 to 5, no single protein or amino acid or glucose was completely deficient or substantially altered to a supraphysiological level. Clearly, pre-implantation embryos require optimal regulation of cellular metabolism via nutrients supplied *in vitro* or *in vivo*^63^. Mouse and human embryos *in vitro* are dependent on glucose and it is required for the blastocyst expansion and hatching, but substantial increased glucose *in vitro* and *in vivo* delayed embryo development and increased embryo death^64^,^65^,^66^,^67^,^68^. Whether or not the altered ULF secretome found in the ULF of PUGKO mice programs the embryo is not known, although GD 4 blastocysts from PUGKO mice exhibited normal implantation in WT uteri. Given that blastocysts from mated PUGKO mice are essentially in developmental delay, it would be interesting to transfer GD 6 or 8 PUGKO blastocysts into GD 4 WT mice to determine their developmental fate.

Collectively, studies of PUGKO and *Foxa2* conditional knockout mice strongly support the hypothesis that secreted factors from the GE are essential for development of uterine receptivity, blastocyst implantation and establishment of pregnancy. Knowledge about the function of most adult GE-specific genes other than LIF is limited. Therefore, the transcriptome and secretome analysis conducted here provides an important framework for future investigations into the biological roles of endometrial glands and their secretions in uterine function, homeostasis, and establishment of pregnancy. These studies are important, as they may lead to critical knowledge important to solve pregnancy loss and complications in women.

## Methods

### Animals

All animal procedures were approved by the Institutional Animal Care and Use Committee of Washington State University and the University of Missouri-Columbia and conducted according to their guidelines for the care and use of laboratory animals. Female PUGKO mice were produced as previously described^18^. Briefly, all C57BL/6 J females in a litter received subcutaneous injections of progesterone (50 μg/g body weight in sesame oil) from postnatal days 2 through 10 (day 0 = birth).

Adult (6–10 week old) C57BL/6 J wildtype and PUGKO females were mated with fertile or vasectomized males to induce pregnancy or pseudopregnancy, respectively. Uteri on days 3, 4, and 5 (day 1 = vaginal plug) were collected for histology and RNA analysis. One uterine horn was fixed in 4% paraformaldehyde in PBS (pH 7.2) at room temperature for 16 h, dehydrated through a series of ethanol and embedded in paraffin for histology. The remaining uterine horn was snap frozen in liquid nitrogen and stored at −80 °C.

Uterine luminal fluid was collected from mice on DOPP 3, 4, and 5 using a previously described method^24^. The female reproductive tract was excised immediately following euthanasia and washed with ice cold PBS, patted dry, and placed under ice cold mineral oil. The cervix and oviduct were removed and retrograde flushing of uterus was performed from the uterotubal junction after 30 μl of sterile PBS was instilled into the uterine lumen. Samples were transferred to a 0.6 ml tube and centrifuged for 5 min at 3,000 × g. The oil-free PBS was stored at −80 °C for proteome, amino acid or glucose analysis.

### Embryo transfer

Wildtype (WT) and PUGKO females were bred to fertile males. Uteri were flushed on day 4 after mating with M2 medium (EMD Millipore) to recover blastocysts. Pseudopregnant recipients were generated by mating females with vasectomized males. Day 4 blastocysts from WT or PUGKO mice were transferred into day 3 uteri of WT or PUGKO psuedopregnant recipients (n = 6 blastocysts per uterine horn). Implantation sites were visualized 72 hours later (day 6) by intravenous injection of 1% Evans blue dye (Sigma-Aldrich Corp., St Louis, MO) into the tail vein 5 min before necropsy.

### Immunohistochemistry

Localization of a specific proteins was performed using a previously described method^18^. Briefly, fixed uteri were sectioned (5 μm), mounted on slides, deparaffinized, and rehydrated in a graded alcohol series. Antigen retrieval was performed by incubating sections for 10 min in boiling 10 mM citrate buffer (pH 6.0). Sections were blocked with 10% normal goat serum in PBS (pH 7.2) and incubated with an antibody to FOXA2 (1.2 μg/ml; LS-C 138006, LifeSpan Biosciences) or MUC1 (1.0 μg/ml; NB120-15481, Novus Biological) overnight at 4 °C. Sections were washed in PBS and incubated with biotinylated secondary antibody (5 μg/ml; Vector Laboratories, Burlingame, CA) for 1 h at 37 °C. Immunoreactive protein was visualized using a Vectastain ABC kit (Vector Laboratories) and diaminobenzidine tetrahydrochloride as the chromagen. Sections were lightly counterstained with hematoxylin before affixing coverslips with Permount.

### Transmission electron microscopy

Uterine horns from DOPP 5 WT and PUGKO mice (n = 3 per type) were fixed in 2% paraformaldehyde/2% glutaraldehyde in 0.1 M cacodylate buffer overnight at 4 °C, rinsed three times in 0.1 M cacodylate buffer and post fixed in 1% OsO4 overnight at 4 °C. Samples were rinsed three times in 0.1 M cacodylate buffer prior to dehydration in an ethanol series, then infiltrated with acetone, embedded in Spurr’s resin and polymerized at 70 °C. Sections were cut to a thickness of 85 nm using an ultramicrotome (Ultracut UCT, Leica Microsystems) and a diamond knife (Diatome). These sections were post-stained using Sato’s triple lead solution stain^69^ and 5% aqueous uranyl acetate. Images were acquired with a JEOL JEM 1400 transmission electron microscope (JEOL) at 80 kV on a Gatan Ultrascan 1000 CCD (Gatan, Inc.) at the Univeristy of Missouri Electron Microscopy Core Facility. After importing into ImageJ 1.49 v, measurements of 10 microvilli from 4 different images were determined for each mouse uterus.

### RNA extraction and real-time PCR

RNA was isolated from uterine samples (n = 4 mice per day and type) using the RNeasy plus mini kit (Qiagen). To eliminate genomic DNA contamination, RNA was treated with DNase I (Qiagen) on-column during RNA isolation. The quantity and purity of total RNA were determined by spectrometry. Total RNA (1 μg) from each sample was reverse transcribed in a total reaction volume of 20 μl using iScript RT supermix (BioRad). Reverse transcription was performed as follows: 5 min at 25 °C; 30 min at 42 °C; and 5 min at 85 °C. Control reactions in the absence of reverse transcriptase were prepared for each sample to test for genomic DNA contamination. The resulting cDNA was stored at −20 °C for further analysis.

Real-time PCR was performed using a CFX384 Touch Real Time System with SsoAdvanced Universal SYBR Green Supermix (BioRad). PrimePCR primers (Bio-Rad) for the quantification of selected genes were used (*Hegf1, Ihh, Lif, Msx1, Noggin, Sfrp4, Wnt7a*). Each sample was analyzed in duplicate with the following conditions for 40 cycles: 95 °C for 30 sec; 95 °C for 5 sec; and 60 °C for 30 sec. A dissociation curve was generated at the end of amplification to ensure that a single product was amplified. PCR without template and template substituted with total RNA were used as negative controls. The threshold line was set in the linear region of the amplification plot above the baseline. Quantification cycle (Cq) values were determined as the cycle number in which the threshold line intersected the amplification curve.

### RNA-sequencing analysis

DOPP 3, 4, and 5 uteri from WT and PUGKO mice (n = 4 mice per treatment) were homogenized in RLT plus buffer and RNA was isolated using RNeasy Plus mini kit (Qiagen). Samples of total RNA (5 μg) were depleted of ribosomal RNA using Ribominus Eukaryote System v2 (Ambion). Ion Total RNA-seq Kit v2 (Life Technologies) was used to construct strand-specific sequencing libraries from ribosomal-depleted samples (20 ng), with several deviations from the protocol. Enzymatic RNA fragmentation was carried out at 37 °C for 90 sec. Fragmented RNA was purified using 1.03 AMPure XP beads (Beckman-Coulter Genomics). The cDNA and final libraries were purified with 0.83 AMPure XP beads. Emulsion PCR was performed on an Ion One Touch 2 instrument (Life Technologies), using the Ion P1 Template OT2 200 v3 reagents. Sequencing beads were quantified and evaluated by flow cytometry using a Guava Easy Cyte (Millipore) with Sybr Gold (Molecular Bioprobes) before loading on an Ion P1 semiconductor sequencing chip. Libraries (n = 4) were sequenced on an Ion Proton using Ion P1 200 v3 sequencing reagents (Life Technologies) at the Washington State University Molecular Biology and Genomics Core. The barcoded libraries were pooled at three per chip for sequencing. Base calling and primary analysis were performed using Torrent Suite 4.0.2 (Life Technologies).

Unmapped BAM files were imported into CLC Genomics Workbench (7.0.4, CLC bio, Aarhus, Denmark). Sequence quality was verified by inspection of quality control reports and samples were processed using CLC Genomics Server (6.0, CLC bio). Reads were quality trimmed (error probability 0.01) with default parameters and mapped to the RefSeq assembly (Mus musculus GRCm38.p1). Alignment BAM files were exported for read quantification of genes using FeatureCounts^70^. Data were deposited in the Gene Expression Omnibus database (GEO, in process).

Differential expression analyses between sample groups were performed by fitting the expression data to a generalized linear model using edgeR-robust^71^. Differentially expressed genes (FDR P < 0.10) were further subjected to weighted correlation network analysis (using R package WGCNA) to identify changes in expression modules (transcriptional networks).

Network analysis were performed using Aracane 2 algorithm. Visualization was done using cytoscape (http://apps.cytoscape.org/apps/aracne). Functional prediction of genes corresponding to those expression modules were assessed by mapping the genes to KEGG (Kyota Encyclopedia of Genes and Genomes) pathways as well GO (Gene Ontology) databases using tools (KEGGscape and BINGO) included in Cytoscape.

Genes that were decreased or absent in PUGKO as compared to WT mice were further analyzed to predict gene functions, pathways and networks. Pathway analysis was performed by topology-based prediction method^38^ implemented in R-bioconductor package ToPAseq^72^. Over-representation of gene-ontology (GO terms) were analyzed using BINGO tool^73^. Biclusters analysis of gene expression was performed using the Order-preserving Submatrix Algorithm (OPSM) which predict large submatrices from expression data^74^ with the BicAT tool^75^.

### Mass spectrometry analysis

Uterine luminal fluid was collected from WT and PUGKO mice on DOPP 3, 4, and 5 (n = 5 mice per treatment). Mass spectrometry analyses were performed by the University of California-Davis Proteomics Center. Proteins were precipitated from the ULF samples using the ProteoExtract Protein Precipitation Kit (Calbiochem). The protein pellet was then solubilized in 100 μL of 6 M urea. Dithiothreitol (DTT; 200 mM) was added to a final concentration of 5 mM, and samples were incubated for 30 min at 37 °C. Next, 20 mM iodoacetamide (IAA) was added to a final concentration of 15 mM and incubated for 30 min at room temp, followed by the addition of 20 μL DTT to quench the IAA. Trypsin/Lys-C (Promega) was added to the sample and incubated for 4 hours at 37 °C. Samples were then diluted to at least 1 M urea by the addition of 50 mM AMBIC and digested overnight at 37 °C. The following day, samples were desalted using Macro Spin Column (Nest Group).

Digested peptides were analyzed by LC-MS/MS on a Thermo Scientific Q Exactive Orbitrap Mass spectrometer in conjunction Proxeon Easy-nLC II HPLC (Thermo Scientific) and Proxeon nanospray source. The digested peptides were loaded a 100 μm × 25 mm Magic C18 100 Å 5U reverse phase trap where they were desalted online before being separated using a 75 μm × 150 mm Magic C18 200 Å 3U reverse phase column. Peptides were eluted using a 90 min gradient with a flow rate of 300 nl per min. An MS survey scan was obtained for the m/z range 300–1600, MS/MS spectra were acquired using a top 15 method, where the top 15 ions in the MS spectra were subjected to HCD (High Energy Collisional Dissociation). An isolation mass window of 2.0 m/z was for the precursor ion selection, and normalized collision energy of 27% was used for fragmentation. A five second duration was used for the dynamic exclusion.

Tandem mass spectra were extracted and charge state deconvoluted by Proteome Discoverer (Thermo Scientific). All MS/MS samples were analyzed using X! Tandem (The GPM, thegpm.org; version TORNADO (2013.02.01.1)). X! Tandem was set up to search *Mus musculus* Proteome database, the cRAP database of common laboratory contaminants (www.thegpm.org/crap; 114 entries) plus an equal number of reverse protein sequences assuming the digestion enzyme trypsin. X! Tandem was searched with a fragment ion mass tolerance of 20 parts per million (ppm) and a parent ion tolerance of 20 ppm. The IAA derivative of cysteine was specified in X! Tandem as a fixed modification. Deamidation of asparagine and glutamine, oxidation of methionine and tryptophan, sulphone of methionine, tryptophan oxidation to formylkynurenin of tryptophan and acetylation of the n-terminus were specified in X! Tandem as variable modifications.

Scaffold (version Scaffold 4.0.6.1, Proteome Software Inc., Portland, OR) was used to validate MS/MS based peptide and protein identifications. Peptide identifications were accepted if they exceeded specific database search engine thresholds. X! Tandem identifications required at least –Log (Expect Scores) scores of greater than 1.2 with a mass accuracy of 5 ppm. Protein identifications were accepted if they contained at least 2 identified peptides. Using the parameters above, the decoy False Discovery Rate (FDR) was calculated to be 1.1% on the protein level and 0.0% on the spectrum level^76^. The minimum number of unique peptides was set at 2 in order for a protein to be identified. Peptide threshold was set at 95% peptide probability, with +2 accepted charge, and parent mass tolerance of 10 parts per million. Further analysis was conducted on peptides identified as exclusive and unique to each protein following the removal of protein clusters. Additional filtering of proteins with 4 or more spectral counts in at least 4 of the 5 samples was applied to compile final lists. Total spectrum counts for proteins were used for comparisons and statistical analysis. FDR was adjusted using the Benjamini-Hochberg procedure to identify significance based on Fisher’s exact test results.

### Glucose analyses

Glucose was quantified in samples of ULF from WT and PUGKO mice on DOPP 3, 4 and 5 (n = 5 mice per day and type) using a fluorometric glucose assay kit (ab65333; Abcam). Each sample was assayed in duplicate. The total recoverable amount of glucose was calculated by multiplying concentrations in the uterine flushing by volume of uterine flushing.

### Amino acid analysis

Amino acids, except for proline, were determined in samples of ULF from WT and PUGKO mice on DOPP 3, 4 and 5 (n = 5 mice per day and type) by fluorometric HPLC methods involving precolumn derivatization with *o*-phthaldialdehyde as previously described for ovine ULF^77^. Briefly, ULF samples were deproteinized with an equal volume of 1.5 M HClO_4_ followed by addition of 2 M K_2_CO_3_. Amino acids in the extract were determined by fluorometric HPLC methods involving precolumn derivatization with *o*-phthaldialdehyde as described previously^78^. The integration of chromatographic peaks was performed using Millenium-32 Software (Waters, Milford, MA). The total recoverable amount of each amino acid was calculated by multiplying concentrations in the uterine flushing by volume of uterine flushing.

### Statistical analysis

All quantitative data was subjected to least-squares analysis of variance using the general linear models procedures of the Statistical Analysis System (SAS Institute) to determine the effects of day, type (WT or PUGKO), and their interaction. In all the analyses, error terms used in tests of significance were identified according to the expectation of the mean squares for error. Significance (P < 0.05) was determined by probability differences of least-squares means. For analysis of real-time PCR data, the Ct values of the target mRNA were analyzed for effects of day, type (WT or PUGKO), and their interaction (day x type) with the *Gapdh, Actb* and *Hprt* values used as covariates. Real-time PCR data are presented as fold change relative to the mRNA level in uteri from the control mice. Probability values < 0.05 were taken to indicate statistical significance.

## Additional Information

**How to cite this article**: Kelleher, A. M. *et al*. Uterine glands impact uterine receptivity, luminal fluid homeostasis and blastocyst implantation. *Sci. Rep.* **6**, 38078; doi: 10.1038/srep38078 (2016).

**Publisher's note:** Springer Nature remains neutral with regard to jurisdictional claims in published maps and institutional affiliations.

## Supplementary Material

Supplemental Figures

Supplemental Table 1

Supplemental Table 2

Supplemental Table 3

Supplemental Table 4

## Figures and Tables

**Table 1:** Top 10 differentially expressed protein coding genes from DOPP 3 to DOPP 4 in the WT uterus.

Gene Symbol	Fold Change	FDR	Mean RPKM	
		P-value	DOPP 3	DOPP 4
*Prss28*	59.86	0.00	1.17	70.22
*Atp6v0d2*	36.31	0.00	0.35	12.69
*8430408G22Rik*	33.16	0.01	0.10	3.35
*Lrat*	32.09	0.00	0.03	0.86
*Prss29*	29.74	0.07	8.42	250.33
*Prss35*	28.95	0.00	0.02	0.70
*Pip*	28.42	0.02	0.25	7.15
*Sval2*	20.09	0.03	0.10	1.95
*Guca2b*	15.81	0.00	0.55	8.72
*Cyp26a1*	15.23	0.00	2.06	31.30
*Zic2*	−3.94	0.03	1.14	0.29
*Vmn2r63*	−4.21	0.07	0.54	0.13
*Spink8*	−4.93	0.04	10.01	2.03
*Sprr2f*	−5.24	0.00	12.71	2.42
*S100a8*	−6.27	0.06	6.23	0.99
*BC048679*	−8.30	0.00	45.51	5.48
*Sprr2g*	−8.42	0.06	1.34	0.16
*S100a9*	−8.71	0.00	16.39	1.88
*Ltf*	−11.57	0.01	120.99	10.45
*Clca3*	−12.97	0.01	9.50	0.73

**Table 2:** Top 10 differentially expressed protein coding genes from DOPP 4 to DOPP 5 in the WT uterus.

Gene Symbol	Fold Change	FDR	Mean RPKM	
		P-value	DOPP 4	DOPP 5
*Gtsf1*	12.56	0.03	0.08	0.97
*Gm10263*	10.55	0.00	17.96	189.48
*Gm21830*	8.80	0.08	0.63	5.51
*Rps18-ps3*	3.74	0.10	8.01	29.97
*Hyal3*	3.33	0.08	0.77	2.58
*Gpr81*	2.92	0.09	0.44	1.27
*C130074G19Rik*	2.59	0.00	11.59	30.04
*H2-Q10*	2.00	0.03	0.52	1.03
*Armcx4*	1.94	0.08	2.02	3.90
*Cd93*	1.86	0.06	10.84	20.17
*Efcab7*	−3.14	0.09	1.16	0.37
*Hist2h2bb*	−3.21	0.07	19.91	6.19
*Naip1*	−3.66	0.02	2.77	0.76
*Fam26e*	−4.00	0.00	1.38	0.35
*Atg16l1*	−4.12	0.03	29.41	7.14
*Catsper1*	−4.86	0.05	0.62	0.13
*Rpl13*	−6.07	0.00	7.23	1.19
*Ccdc107*	−6.19	0.00	251.44	40.65
*1700020N01Rik*	−11.14	0.03	76.26	6.84
*Npffr2*	−14.21	0.09	4.23	0.30

**Table 3:** Top 10 Gene ontology (GO) terms enriched in genes absent or decreased in the uterus of WT as compared to PUGKO mice.

GO-ID	Process	P-value	Description
6775	fat-soluble vitamin metabolic process	1.76E-06	Absent in PUGKO uterus
65008	regulation of biological quality	2.11E-06	Absent in PUGKO uterus
6776	vitamin A metabolic process	1.36E-05	Absent in PUGKO uterus
1523	retinoid metabolic process	2.22E-05	Absent in PUGKO uterus
16101	diterpenoid metabolic process	2.22E-05	Absent in PUGKO uterus
6721	terpenoid metabolic process	2.57E-05	Absent in PUGKO uterus
42445	hormone metabolic process	4.00E-05	Absent in PUGKO uterus
34754	cellular hormone metabolic process	4.15E-05	Absent in PUGKO uterus
55114	oxidation reduction	4.80E-05	Absent in PUGKO uterus
10817	regulation of hormone levels	5.07E-05	Absent in PUGKO uterus
9987	cellular process	1.62E-11	Decreased in PUGKO uterus
65008	regulation of biological quality	8.19E-11	Decreased in PUGKO uterus
6810	transport	1.35E-10	Decreased in PUGKO uterus
51234	establishment of localization	1.91E-10	Decreased in PUGKO uterus
8152	metabolic process	2.82E-10	Decreased in PUGKO uterus
51179	localization	3.72E-10	Decreased in PUGKO uterus
9611	response to wounding	6.01E-10	Decreased in PUGKO uterus
55114	oxidation reduction	2.20E-08	Decreased in PUGKO uterus
50878	regulation of body fluid levels	2.61E-08	Decreased in PUGKO uterus
50896	response to stimulus	5.52E-08	Decreased in PUGKO uterus

**Table 4:** Most abundant proteins identified in WT ULF.

Protein Description	Accession Number	Molecular Weight	Average Spectral Counts		
			DOPP 3	DOPP 4	DOPP 5
Serum albumin	sp|P07724|ALBU_MOUSE	69 kDa	673.4	281	286.4
Beta-globin	tr|A8DUK4|A8DUK4_MOUSE	16 kDa	385.4	217	185.6
Hemoglobin subunit beta-1	sp|P02088|HBB1_MOUSE	16 kDa	273.2	135.8	136
Alpha globin 1	tr|Q91VB8|Q91VB8_MOUSE	15 kDa	234	202	172.8
Hemoglobin subunit alpha	sp|P01942|HBA_MOUSE	15 kDa	204	179.8	156.4
Hemoglobin subunit beta-2	sp|P02089|HBB2_MOUSE	16 kDa	154.2	80.4	81.8
Serotransferrin	sp|Q921I1|TRFE_MOUSE	77 kDa	134.4	83.8	75.8
Protein Gm20425	tr|E9Q035|E9Q035_MOUSE	108 kDa	127.2	78.6	71.4
Hemoglobin subunit alpha	sp|P69905|HBA_HUMAN	15 kDa	75.6	26.8	39.4
Actin, cytoplasmic 1	sp|P60710|ACTB_MOUSE	42 kDa	75.4	64	50.4
Hemoglobin subunit beta	sp|P68871|HBB_HUMAN	16 kDa	68.4	42.4	27.8
Alpha-2-macroglobulin	sp|Q61838|A2M_MOUSE	166 kDa	64	35.2	25.6
Complement C3	sp|P01027|CO3_MOUSE	186 kDa	56.8	30.8	29.4
Myosin-9	sp|Q8VDD5|MYH9_MOUSE	226 kDa	56.4	54.4	58.2
Hemoglobin subunit epsilon-Y2	sp|P02104|HBE_MOUSE	16 kDa	53.6	34.6	17
Tubulin beta-5 chain	sp|P99024|TBB5_MOUSE	50 kDa	49	19.6	15.2
Actin, alpha skeletal muscle	sp|P68134|ACTS_MOUSE	42 kDa	47	48.2	37.2
Heat shock cognate 71 kDa protein	sp|P63017|HSP7C_MOUSE	71 kDa	46	31.6	30
Tubulin beta-4B chain	sp|P68372|TBB4B_MOUSE	50 kDa	45.4	16	12
Actin, aortic smooth muscle	sp|P62737|ACTA_MOUSE	42 kDa	44.8	47.4	35.8

**Table 5:** Amino Acid Concentrations [nmol/ml] in the ULF of WT mice.

Amino Acid	DOPP 3	DOPP 4	DOPP 5
Ala	20.7 ± 1.7	26.8 ± 2.9	25.1 ± 1.5
Arg	4.6 ± 0.4	7.9 ± 0.8^a^	8.1 ± 1.1^a^
Asn	3.8 ± 0.3	5.7 ± 0.7^a^	5.5 ± 0.5^a^
Asp	15.7 ± 0.9	21.3 ± 1.9^a^	19.2 ± 1.1
b-Ala	4.2 ± 0.7	16.3 ± 0.8^a^	10.7 ± 0.8^ab^
Cys	1.4 ± 0.1	2.3 ± 0.3^a^	1.6 ± 0.1^b^
Gln	13.3 ± 1.1	21.2 ± 2.5^a^	20.1 ± 2.7^a^
Glu	18.4 ± 2.1	29.4 ± 3.8^a^	25.6 ± 2.6
Gly	38.3 ± 2.9	65.1 ± 5.7^a^	58.0 ± 3.1^a^
His	3.3 ± 0.7	5.1 ± 0.5	4.2 ± 0.7
Ile	7.8 ± 1.0	9.1 ± 0.4	7.6 ± 0.3
Leu	34.4 ± 2.3	18.3 ± 1.5^a^	23.2 ± 0.8^ab^
Lys	13.7 ± 0.2	16.6 ± 1.0^a^	15.7 ± 1.1
Met	5.2 ± 0.4	8.5 ± 0.3^a^	6.7 ± 0.4^ab^
Orn	40.1 ± 0.2	49.3 ± 1.5^a^	45.1 ± 1.6^ab^
Phe	18.9 ± 0.3	16.9 ± 0.7^a^	17.4 ± 0.2^a^
Ser	27.3 ± 0.6	39.8 ± 4.4^a^	33.7 ± 1.3
Tau	91.4 ± 10.3	152.4 ± 25.7	142.1 ± 23.4
Thr	6.5 ± 0.5	12.5 ± 1.8^a^	9.8 ± 0.6
Trp	5.7 ± 0.4	7.6 ± 0.3^a^	6.7 ± 0.2^ab^
Tyr	24.2 ± 0.8	17.1 ± 0.9^a^	20.0 ± 0.5^ab^
Val	13.3 ± 0.5	16.5 ± 1.2^a^	14.8 ± 0.6

Different superscripts indicate significant differences between days (*P* < 0.05).

^a^Denotes a difference from DOPP 3 vs DOPP 4.

^b^Denotes a difference from DOPP 4 vs DOPP 5.

## References

[b1] Dey S. K. . Molecular cues to implantation. Endocr Rev. 25, 341–373 (2004). 15180948 10.1210/er.2003-0020

[b2] Cha J., Sun X. & Dey S. K. Mechanisms of implantation: strategies for successful pregnancy. Nat Med. 18, 1754–1767 (2012). 23223073 10.1038/nm.3012 PMC6322836

[b3] Burton G. J., Jauniaux E. & Charnock-Jones D. S. Human early placental development: potential roles of the endometrial glands. Placenta. 28 Suppl A, S64–69 (2007). 17349689 10.1016/j.placenta.2007.01.007 PMC1878510

[b4] Dimitriadis E. . Interleukin-11, IL-11 receptoralpha and leukemia inhibitory factor are dysregulated in endometrium of infertile women with endometriosis during the implantation window. J Reprod Immunol. 69, 53–64 (2006). 16310857 10.1016/j.jri.2005.07.004

[b5] Berlanga O., Bradshaw H. B., Vilella-Mitjana F., Garrido-Gomez T. & Simon C. How endometrial secretomics can help in predicting implantation. Placenta. 32 Suppl 3, S271–275 (2011). 21700334 10.1016/j.placenta.2011.06.002

[b6] Wang H. & Dey S. K. Roadmap to embryo implantation: clues from mouse models. Nature reviews. Genetics. 7, 185–199 (2006). 10.1038/nrg1808 16485018

[b7] Carson D. D. . Embryo implantation. Developmental biology. 223, 217–237 (2000). 10882512 10.1006/dbio.2000.9767

[b8] Enders A. C. & Schlafke S. Comparative aspects of blastocyst-endometrial interactions at implantation. Ciba Found Symp, 3–32 (1978). 10.1002/9780470720479.ch2 115657

[b9] Schlafke S. & Enders A. C. Cellular basis of interaction between trophoblast and uterus at implantation. Biology of reproduction. 12, 41–65 (1975). 1095088 10.1095/biolreprod12.1.41

[b10] Ramathal C. Y., Bagchi I. C., Taylor R. N. & Bagchi M. K. Endometrial decidualization: of mice and men. Semin Reprod Med. 28, 17–26 (2010). 20104425 10.1055/s-0029-1242989 PMC3095443

[b11] Wetendorf M. & DeMayo F. J. The progesterone receptor regulates implantation, decidualization, and glandular development via a complex paracrine signaling network. Mol Cell Endocrinol. 357, 108–118 (2012). 22115959 10.1016/j.mce.2011.10.028 PMC3443857

[b12] Bhatt H., Brunet L. J. & Stewart C. L. Uterine expression of leukemia inhibitory factor coincides with the onset of blastocyst implantation. Proc Natl Acad Sci USA 88, 11408–11412 (1991). 1722331 10.1073/pnas.88.24.11408 PMC53144

[b13] Das S. K., Tsukamura H., Paria B. C., Andrews G. K. & Dey S. K. Differential expression of epidermal growth factor receptor (EGF-R) gene and regulation of EGF-R bioactivity by progesterone and estrogen in the adult mouse uterus. Endocrinology. 134, 971–981 (1994). 7507841 10.1210/endo.134.2.7507841

[b14] Huet-Hudson Y. M. . Estrogen regulates the synthesis of epidermal growth factor in mouse uterine epithelial cells. Mol Endocrinol. 4, 510–523 (1990). 2342484 10.1210/mend-4-3-510

[b15] Gray C. A., Burghardt R. C., Johnson G. A., Bazer F. W. & Spencer T. E. Evidence that absence of endometrial gland secretions in uterine gland knockout ewes compromises conceptus survival and elongation. Reproduction. 124, 289–300 (2002). 12141942

[b16] Gray C. A. . Endometrial glands are required for preimplantation conceptus elongation and survival. Biology of reproduction. 64, 1608–1613 (2001). 11369585 10.1095/biolreprod64.6.1608

[b17] Filant J. & Spencer T. E. Endometrial glands are essential for blastocyst implantation and decidualization in the mouse uterus. Biology of reproduction. 88, 93 (2013). 23407384 10.1095/biolreprod.113.107631

[b18] Filant J., Zhou H. & Spencer T. E. Progesterone inhibits uterine gland development in the neonatal mouse uterus. Biology of reproduction. 86, 146, 141–149 (2012). 22238285 10.1095/biolreprod.111.097089 PMC3364926

[b19] Cooke P. S. . Brief exposure to progesterone during a critical neonatal window prevents uterine gland formation in mice. Biology of reproduction. 86, 63 (2012). 22133692 10.1095/biolreprod.111.097188 PMC3316263

[b20] Hempstock J., Cindrova-Davies T., Jauniaux E. & Burton G. Endometrial glands as a source of nutrients, growth factors and cytokines during the first trimester of human pregnancy: A morphological and immunohistochemical study. Reproductive Biology and Endocrinology. 2, 58 (2004). 15265238 10.1186/1477-7827-2-58 PMC493283

[b21] Hannan N. J. . 2D-DiGE analysis of the human endometrial secretome reveals differences between receptive and nonreceptive states in fertile and infertile women. J Proteome Res. 9, 6256–6264 (2010). 20925431 10.1021/pr1004828

[b22] Spencer T. E., Sandra O. & Wolf E. Genes involved in conceptus-endometrial interactions in ruminants: insights from reductionism and thoughts on holistic approaches. Reproduction. 135, 165–179 (2008). 18239047 10.1530/REP-07-0327

[b23] Koch J. M., Ramadoss J. & Magness R. R. Proteomic profile of uterine luminal fluid from early pregnant ewes. J Proteome Res. 9, 3878–3885 (2010). 20578732 10.1021/pr100096b PMC3124775

[b24] Harris S. E., Gopichandran N., Picton H. M., Leese H. J. & Orsi N. M. Nutrient concentrations in murine follicular fluid and the female reproductive tract. Theriogenology. 64, 992–1006 (2005). 16054501 10.1016/j.theriogenology.2005.01.004

[b25] Stewart C. L. . Blastocyst implantation depends on maternal expression of leukaemia inhibitory factor. Nature. 359, 76–79 (1992). 1522892 10.1038/359076a0

[b26] Dunlap K. A. . Postnatal deletion of Wnt7a inhibits uterine gland morphogenesis and compromises adult fertility in mice. Biology of reproduction. 85, 386–396 (2011). 21508348 10.1095/biolreprod.111.091769 PMC3142262

[b27] Jeong J. W. . Foxa2 is essential for mouse endometrial gland development and fertility. Biology of reproduction. 83, 396–403 (2010). 20484741 10.1095/biolreprod.109.083154 PMC2924802

[b28] Franco H. L. . WNT4 is a key regulator of normal postnatal uterine development and progesterone signaling during embryo implantation and decidualization in the mouse. FASEB J. 25, 1176–1187 (2011). 21163860 10.1096/fj.10-175349 PMC3058697

[b29] Cooke P. S. . Uterine gland development begins postnatally and is accompanied by estrogen and progesterone receptor expression in the dog. Theriogenology. 78, 1787–1795 (2012). 22959316 10.1016/j.theriogenology.2012.05.028

[b30] Filant J., Lydon J. P. & Spencer T. E. Integrated chromatin immunoprecipitation sequencing and microarray analysis identifies FOXA2 target genes in the glands of the mouse uterus. FASEB J. 28, 230–243 (2014). 24025729 10.1096/fj.13-237446 PMC7092800

[b31] Psychoyos A. Hormonal control of ovoimplantation. Vitam Horm. 31, 201–256 (1973). 4620375 10.1016/s0083-6729(08)60999-1

[b32] Monsivais D. . Uterine ALK3 is essential during the window of implantation. Proc Natl Acad Sci USA 113, E387–395 (2016). 26721398 10.1073/pnas.1523758113 PMC4725477

[b33] Brayman M., Thathiah A. & Carson D. D. MUC1: a multifunctional cell surface component of reproductive tissue epithelia. Reprod Biol Endocrinol. 2, 4 (2004). 14711375 10.1186/1477-7827-2-4 PMC320498

[b34] Murphy C. R. Understanding the apical surface markers of uterine receptivity: pinopods-or uterodomes? Hum Reprod. 15, 2451–2454 (2000). 11098008 10.1093/humrep/15.12.2451

[b35] Lee K. Y., Jeong J. W., Tsai S. Y., Lydon J. P. & DeMayo F. J. Mouse models of implantation. Trends Endocrinol Metab. 18, 234–239 (2007). 17588769 10.1016/j.tem.2007.06.002

[b36] Deb K., Reese J. & Paria B. C. InPlacenta and Trophoblast: Methods and Protocols Vol. 1 Methods in Molecular Medicine (eds Soares M. J. & Hunt J. S.) Ch. 2, 9–34 (Humana Press, 2005).

[b37] Filant J. & Spencer T. E. Cell-specific transcriptional profiling reveals candidate mechanisms regulating development and function of uterine epithelia in mice. Biology of reproduction. 89, 86 (2013). 23946541 10.1095/biolreprod.113.111971 PMC7289334

[b38] Ibrahim M. A., Jassim S., Cawthorne M. A. & Langlands K. A topology-based score for pathway enrichment. J Comput Biol. 19, 563–573 (2012). 22468678 10.1089/cmb.2011.0182

[b39] Martin K. L. & Leese H. J. Role of glucose in mouse preimplantation embryo development. Mol Reprod Dev. 40, 436–443 (1995). 7598909 10.1002/mrd.1080400407

[b40] Frolova A. I. & Moley K. H. Glucose transporters in the uterus: an analysis of tissue distribution and proposed physiological roles. Reproduction. 142, 211–220 (2011). 21642384 10.1530/REP-11-0114 PMC5028205

[b41] Martin P. M., Sutherland A. E. & Van Winkle L. J. Amino acid transport regulates blastocyst implantation. Biology of reproduction. 69, 1101–1108 (2003). 12801981 10.1095/biolreprod.103.018010

[b42] Gonzalez I. M. . Leucine and arginine regulate trophoblast motility through mTOR-dependent and independent pathways in the preimplantation mouse embryo. Developmental biology. 361, 286–300 (2012). 22056783 10.1016/j.ydbio.2011.10.021 PMC3246567

[b43] Jeong J. W. . beta-catenin mediates glandular formation and dysregulation of beta-catenin induces hyperplasia formation in the murine uterus. Oncogene. 28, 31–40 (2009). 18806829 10.1038/onc.2008.363 PMC2646831

[b44] Yu X. . Activation of beta-Catenin in mouse prostate causes HGPIN and continuous prostate growth after castration. Prostate. 69, 249–262 (2009). 18991257 10.1002/pros.20877 PMC4437562

[b45] DeSouza M. M. . MUC1/episialin: a critical barrier in the female reproductive tract. J Reprod Immunol. 45, 127–158 (1999). 10674981 10.1016/s0165-0378(99)00046-7

[b46] Denker H. W. Implantation: a cell biological paradox. J Exp Zool. 266, 541–558 (1993). 8371097 10.1002/jez.1402660606

[b47] Fouladi-Nashta A. A. . Characterization of the uterine phenotype during the peri-implantation period for LIF-null, MF1 strain mice. Developmental biology. 281, 1–21 (2005). 15848385 10.1016/j.ydbio.2005.01.033

[b48] Chen J. R. . Leukemia inhibitory factor can substitute for nidatory estrogen and is essential to inducing a receptive uterus for implantation but is not essential for subsequent embryogenesis. Endocrinology. 141, 4365–4372 (2000). 11108244 10.1210/endo.141.12.7855

[b49] Chen W., Han B. C., Wang R. C., Xiong G. F. & Peng J. P. Role of secretory protease inhibitor SPINK3 in mouse uterus during early pregnancy. Cell Tissue Res. 341, 441–451 (2010). 20623140 10.1007/s00441-010-1013-5

[b50] Stocco D. M. A review of the characteristics of the protein required for the acute regulation of steroid hormone biosynthesis: the case for the steroidogenic acute regulatory (StAR) protein. Proc Soc Exp Biol Med. 217, 123–129 (1998). 9452135 10.3181/00379727-217-44214

[b51] Stocco D. M. StAR protein and the regulation of steroid hormone biosynthesis. Annu Rev Physiol. 63, 193–213 (2001). 11181954 10.1146/annurev.physiol.63.1.193

[b52] Jeong J. W. . Identification of murine uterine genes regulated in a ligand-dependent manner by the progesterone receptor. Endocrinology. 146, 3490–3505 (2005). 15845616 10.1210/en.2005-0016

[b53] Deng L. . Coordinate regulation of the production and signaling of retinoic acid by estrogen in the human endometrium. J Clin Endocrinol Metab. 88, 2157–2163 (2003). 12727970 10.1210/jc.2002-021844

[b54] Vermot J., Fraulob V., Dolle P. & Niederreither K. Expression of enzymes synthesizing (aldehyde dehydrogenase 1 and reinaldehyde dehydrogenase 2) and metabolizaing (Cyp26) retinoic acid in the mouse female reproductive system. Endocrinology. 141, 3638–3645 (2000). 11014218 10.1210/endo.141.10.7696

[b55] Ponnamperuma R. M., Kirchhof S. M., Trifiletti L. & De Luca L. M. Ovariectomy increases squamous metaplasia of the uterine horns and survival of SENCAR mice fed a vitamin A-deficient diet. Am J Clin Nutr. 70, 502–508 (1999). 10500019 10.1093/ajcn/70.4.502

[b56] Bruner-Tran K. L. . Steroid and cytokine regulation of matrix metalloproteinase expression in endometriosis and the establishment of experimental endometriosis in nude mice. J Clin Endocrinol Metab. 87, 4782–4791 (2002). 12364474 10.1210/jc.2002-020418

[b57] Curry T. E. Jr. & Osteen K. G. The matrix metalloproteinase system: changes, regulation, and impact throughout the ovarian and uterine reproductive cycle. Endocr Rev. 24, 428–465 (2003). 12920150 10.1210/er.2002-0005

[b58] Hediger M. A., Clemencon B., Burrier R. E. & Bruford E. A. The ABCs of membrane transporters in health and disease (SLC series): introduction. Mol Aspects Med. 34, 95–107 (2013). 23506860 10.1016/j.mam.2012.12.009 PMC3853582

[b59] Martin P. M. & Sutherland A. E. Exogenous amino acids regulate trophectoderm differentiation in the mouse blastocyst through an mTOR-dependent pathway. Developmental biology. 240, 182–193 (2001). 11784055 10.1006/dbio.2001.0461

[b60] Spencer T. E. Biological roles of uterine glands in pregnancy. Semin Reprod Med. 32, 346–357 (2014). 24959816 10.1055/s-0034-1376354 PMC4198167

[b61] Bazer F. W. . Select nutrients in the uterine lumen of sheep and pigs affect conceptus development. J Reprod Dev. 58, 180–188 (2012). 22738901 10.1262/jrd.2011-019

[b62] Spencer T. E. & Hansen T. R. Implantation and Establishment of Pregnancy in Ruminants. Adv Anat Embryol Cell Biol. 216, 105–135 (2015). 26450497 10.1007/978-3-319-15856-3_7

[b63] Leese H. J. Metabolism of the preimplantation embryo: 40 years on. Reproduction. 143, 417–427 (2012). 22408180 10.1530/REP-11-0484

[b64] Ramin N. . Maternal diabetes impairs gastrulation and insulin and IGF-I receptor expression in rabbit blastocysts. Endocrinology. 151, 4158–4167 (2010). 20631000 10.1210/en.2010-0187

[b65] Moley K. H., Vaughn W. K., DeCherney A. H. & Diamond M. P. Effect of diabetes mellitus on mouse pre-implantation embryo development. Journal of reproduction and fertility. 93, 325–332 (1991). 1787451 10.1530/jrf.0.0930325

[b66] Vercheval M. . Experimental diabetes impairs rat embryo development during the preimplantation period. Diabetologia. 33, 187–191 (1990). 2347432 10.1007/BF00404794

[b67] Pantaleon M., Tan H. Y., Kafer G. R. & Kaye P. L. Toxic effects of hyperglycemia are mediated by the hexosamine signaling pathway and o-linked glycosylation in early mouse embryos. Biology of reproduction. 82, 751–758 (2010). 20032283 10.1095/biolreprod.109.076661 PMC2842489

[b68] Wyman A., Pinto A. B., Sheridan R. & Moley K. H. One-cell zygote transfer from diabetic to nondiabetic mouse results in congenital malformations and growth retardation in offspring. Endocrinology. 149, 466–469 (2008). 18039778 10.1210/en.2007-1273 PMC2219313

[b69] Sato T. A modified method for lead staining of thin sections. J Electron Microsc (Tokyo). 17, 158–159 (1968). 4177281

[b70] Liao Y., Smyth G. K. & Shi W. featureCounts: an efficient general purpose program for assigning sequence reads to genomic features. Bioinformatics. 30, 923–930 (2014). 24227677 10.1093/bioinformatics/btt656

[b71] Zhou X., Lindsay H. & Robinson M. D. Robustly detecting differential expression in RNA sequencing data using observation weights. Nucleic Acids Res. 42, e91 (2014). 24753412 10.1093/nar/gku310 PMC4066750

[b72] Ihnatova I. & Budinska E. ToPASeq: an R package for topology-based pathway analysis of microarray and RNA-Seq data. BMC Bioinformatics. 16, 350 (2015). 26514335 10.1186/s12859-015-0763-1 PMC4625615

[b73] Maere S., Heymans K. & Kuiper M. BiNGO: a Cytoscape plugin to assess overrepresentation of gene ontology categories in biological networks. Bioinformatics. 21, 3448–3449 (2005). 15972284 10.1093/bioinformatics/bti551

[b74] Ben-Dor A., Chor B., Karp R. & Yakhini Z. Discovering local structure in gene expression data: the order-preserving submatrix problem. J Comput Biol. 10, 373–384 (2003). 12935334 10.1089/10665270360688075

[b75] Barkow S., Bleuler S., Prelic A., Zimmermann P. & Zitzler E. BicAT: a biclustering analysis toolbox. Bioinformatics. 22, 1282–1283 (2006). 16551664 10.1093/bioinformatics/btl099

[b76] Tabb D. L. What’s driving false discovery rates? J Proteome Res. 7, 45–46 (2008). 18081243 10.1021/pr700728t PMC2810656

[b77] Gao H., Wu G., Spencer T. E., Johnson G. A. & Bazer F. W. Select nutrients in the ovine uterine lumen. IV. Expression of neutral and acidic amino acid transporters in ovine uteri and peri-implantation conceptuses. Biology of reproduction. 80, 1196–1208 (2009). 19176878 10.1095/biolreprod.108.075440

[b78] Wu G., Davis P. K., Flynn N. E., Knabe D. A. & Davidson J. T. Endogenous synthesis of arginine plays an important role in maintaining arginine homeostasis in postweaning growing pigs. J Nutr. 127, 2342–2349 (1997). 9405584 10.1093/jn/127.12.2342

