# Supplementary material for: Uterine glands impact uterine receptivity, luminal fluid homeostasis and blastocyst implantation
Source: Sci Rep. 2016 Dec 1;6:38078. doi: 10.1038/srep38078 (PMC5131473; doi:10.1038/srep38078)
Supplement: Supplemental Figures [file srep38078-s1.pdf]

Uterine glands impact uterine receptivity, luminal fluid homeostasis and blastocyst implantation

Andrew M. Kelleher[1], Gregory W. Burns[1], Susanta Behura[1], Guoyao Wu[2] & Thomas E. Spencer[1*]

Supplemental Figure Legends

Supplemental Figure 1. Coagulation and complement cascades pathway. This KEGG pathway denotes differentially expressed genes absent in PUGKO as compared to WT mouse uterus. The genes shown in yellow/red are predicted to perturb the pathway.

Supplemental Figure 2. Predicted gene networks in the uterus of WT and PUGKO mice. **A-B)** Predicted networks of genes that are expressed in WT uteri but absent in knockout mice. **C-D)** Predicted networks of genes that are expressed in WT uteri but decreased in PUGKO mice.

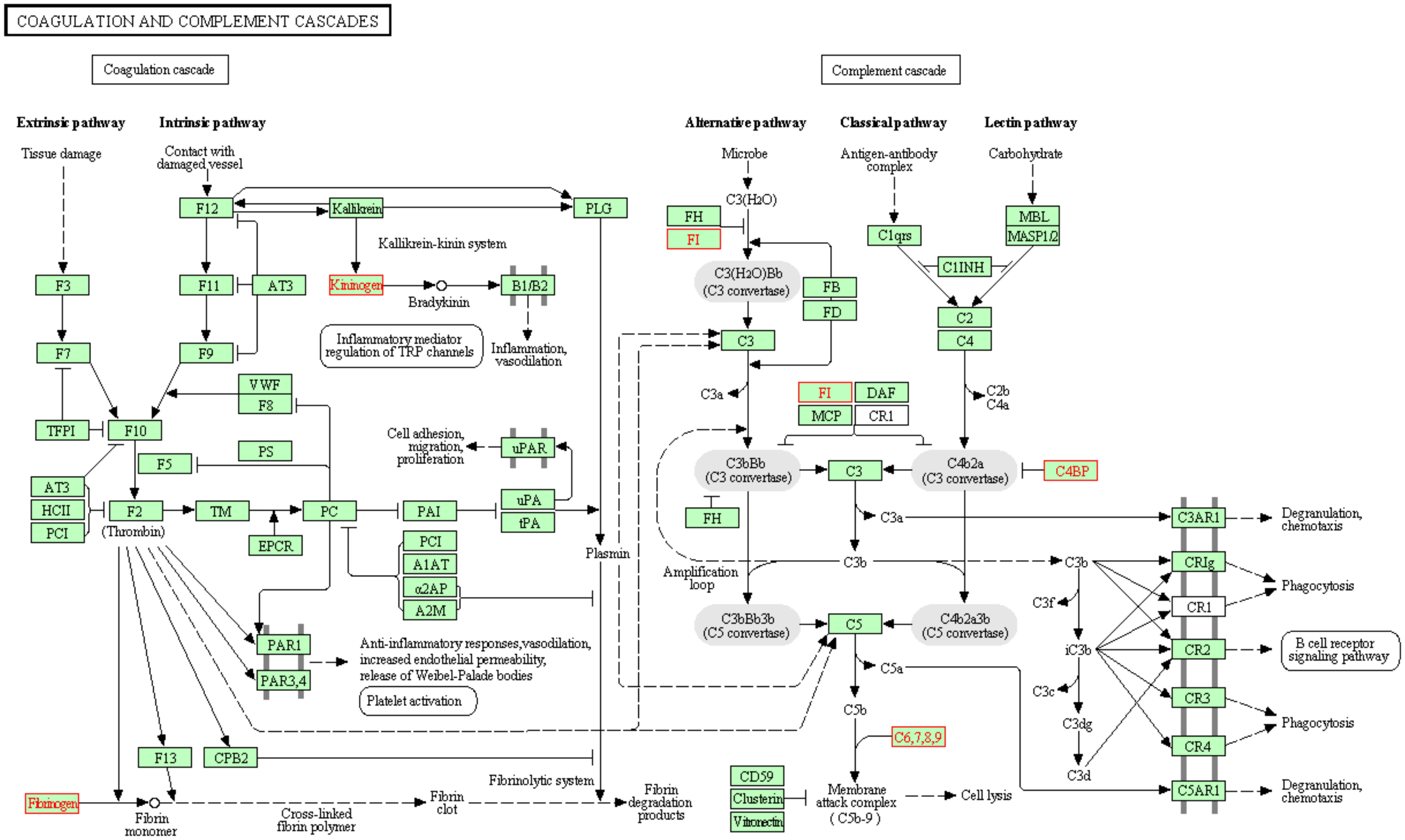
COAGULATION AND COMPLEMENT CASCADES
Coagulation cascade
Complement cascade
Extrinsic pathway
Intrinsic pathway
Alternative pathway
Classical pathway
Lectin pathway
Tissue damage
Contact with damaged vessel
Microbe
Antigen-antibody complex
Carbohydrate
F12
Kallikrein
PLG
F3
F11
AT3
Kininogen
Bradykinin
B1/B2
Kallikrein-kinin system
F7
F9
Inflammatory mediator regulation of TRP channels
Inflammation, vasodilation
VWF
F8
TFPI
F10
PS
F5
Cell adhesion, migration, proliferation
uPAR
AT3
HCII
PCI
F2
(Thrombin)
TM
PC
EPCR
PAI
uPA
tPA
Plasmin
PCI
A1AT
α2AP
A2M
PAR1
PAR3,4
Anti-inflammatory responses, vasodilation, increased endothelial permeability, release of Weibel-Palade bodies
Platelet activation
F13
CPB2
Fibrinolytic system
Fibrinogen
Fibrin monomer
Cross-linked fibrin polymer
Fibrin clot
Fibrin degradation products
FH
FI
C3(H2O)
C3(H2O)Bb (C3 convertase)
FB
FD
C3
C1qrs
MBL
MASP1/2
C1INH
C2
C4
C3a
FI
DAF
MCP
CR1
C2b
C4a
C3bBb (C3 convertase)
C3
C4b2a (C3 convertase)
C4BP
FH
C3a
C3AR1
Degranulation, chemotaxis
Amplification loop
C3b
C3b
CRIg
Phagocytosis
C3f
CR1
C3bBb3b (C5 convertase)
C5
C4b2a3b (C5 convertase)
iC3b
CR2
B cell receptor signaling pathway
C5a
C3c
CR3
Phagocytosis
C5b
C3dg
CR4
C6,7,8,9
CD59
Clusterin
Vitronectin
Membrane attack complex (C5b-9)
Cell lysis
C3d
C5AR1
Degranulation, chemotaxis

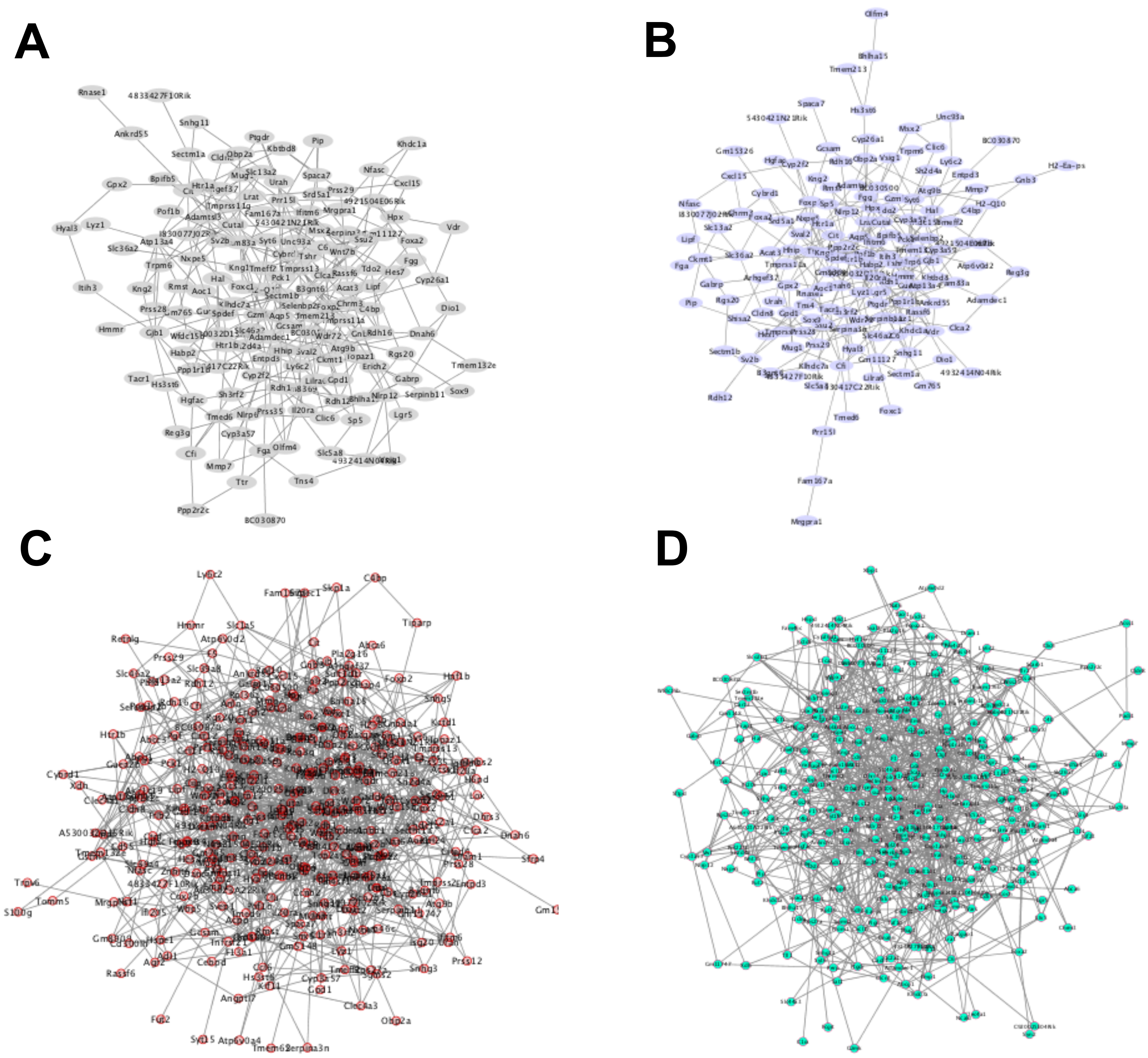
A
B
C
D
